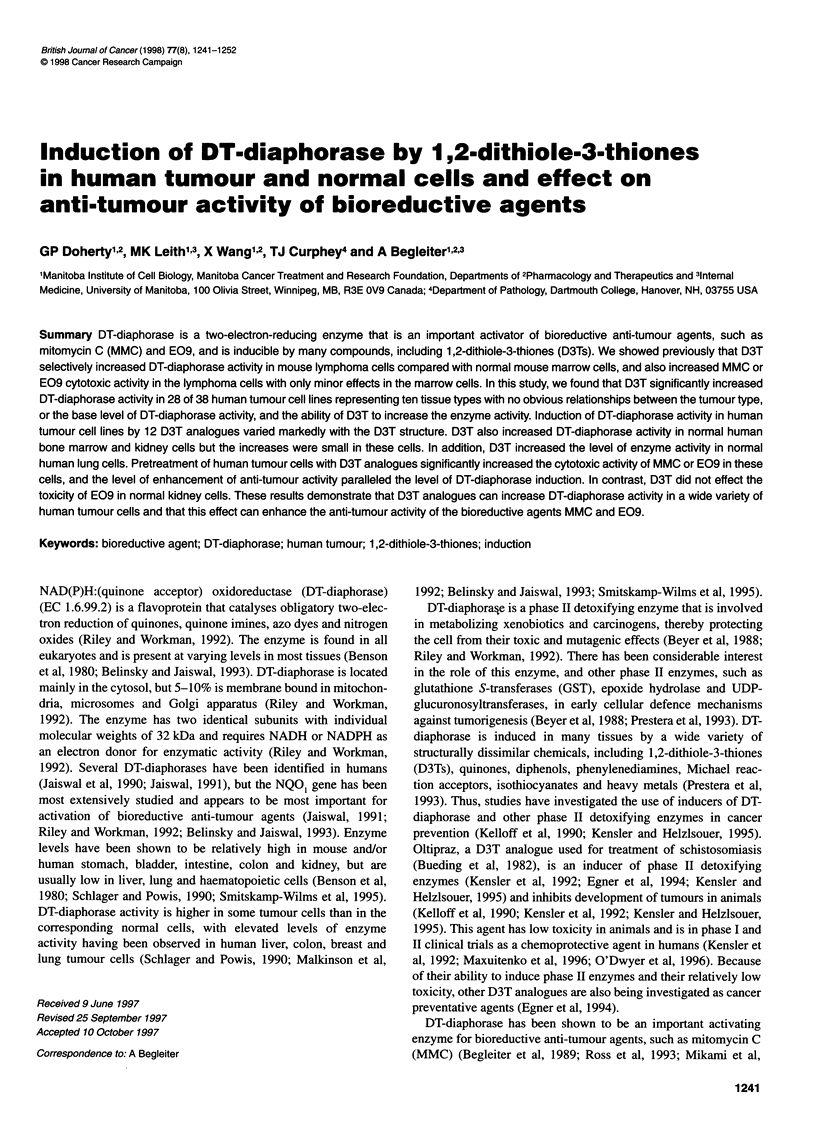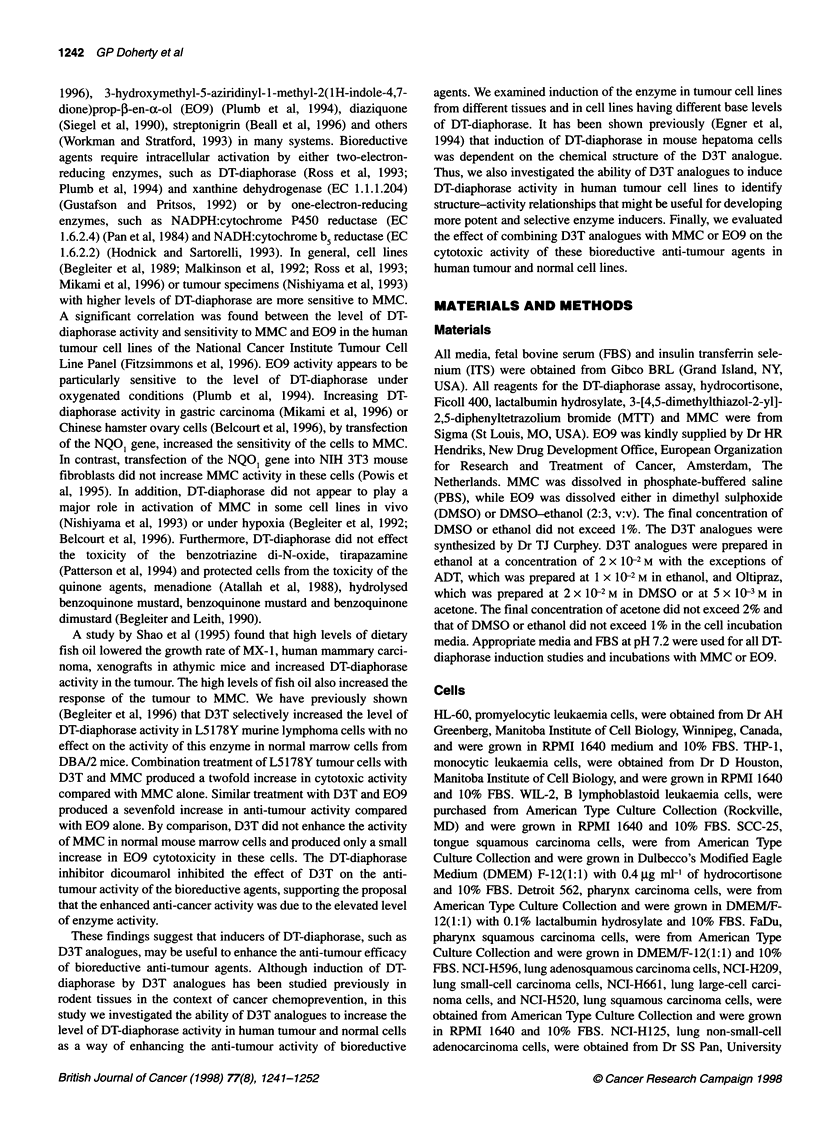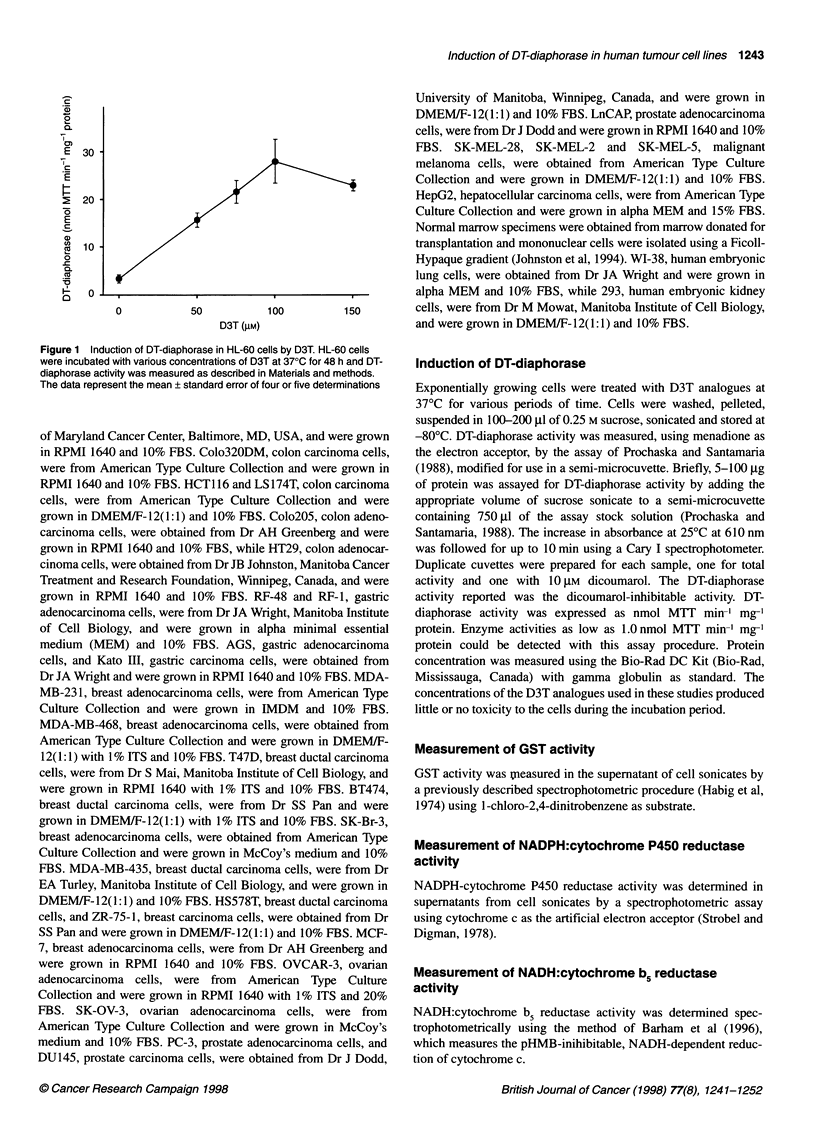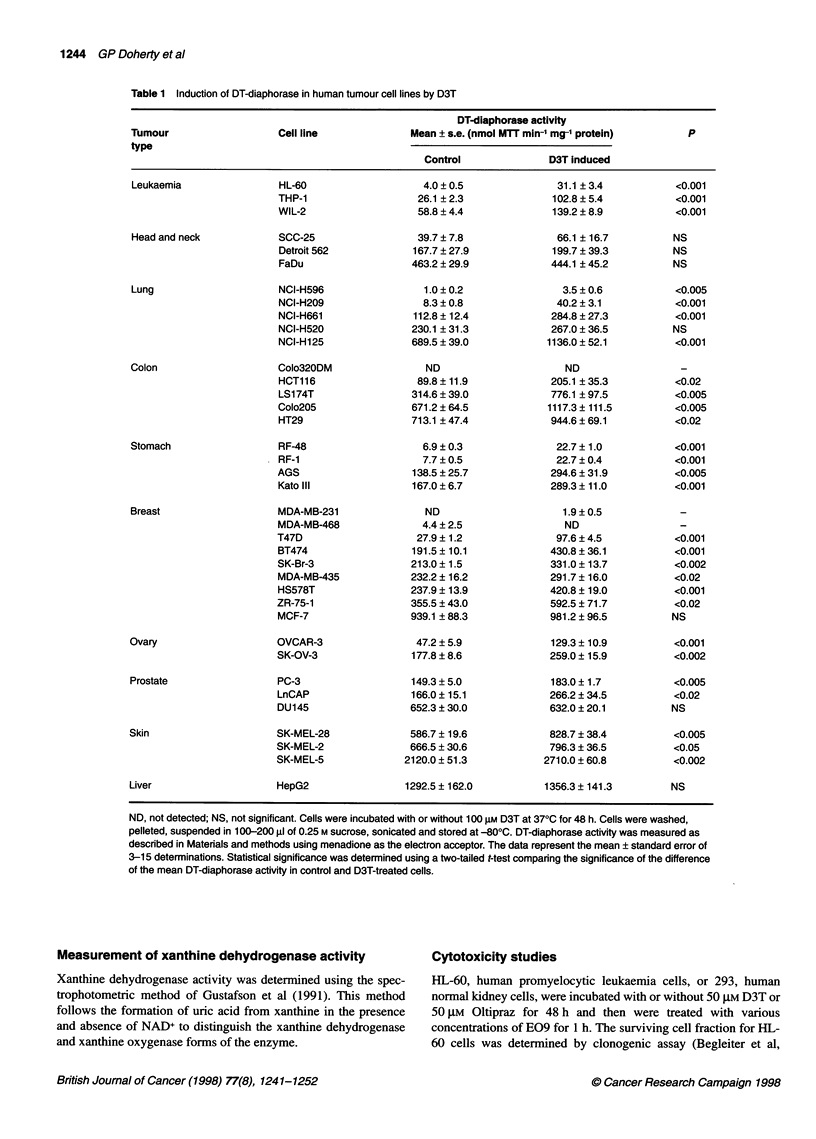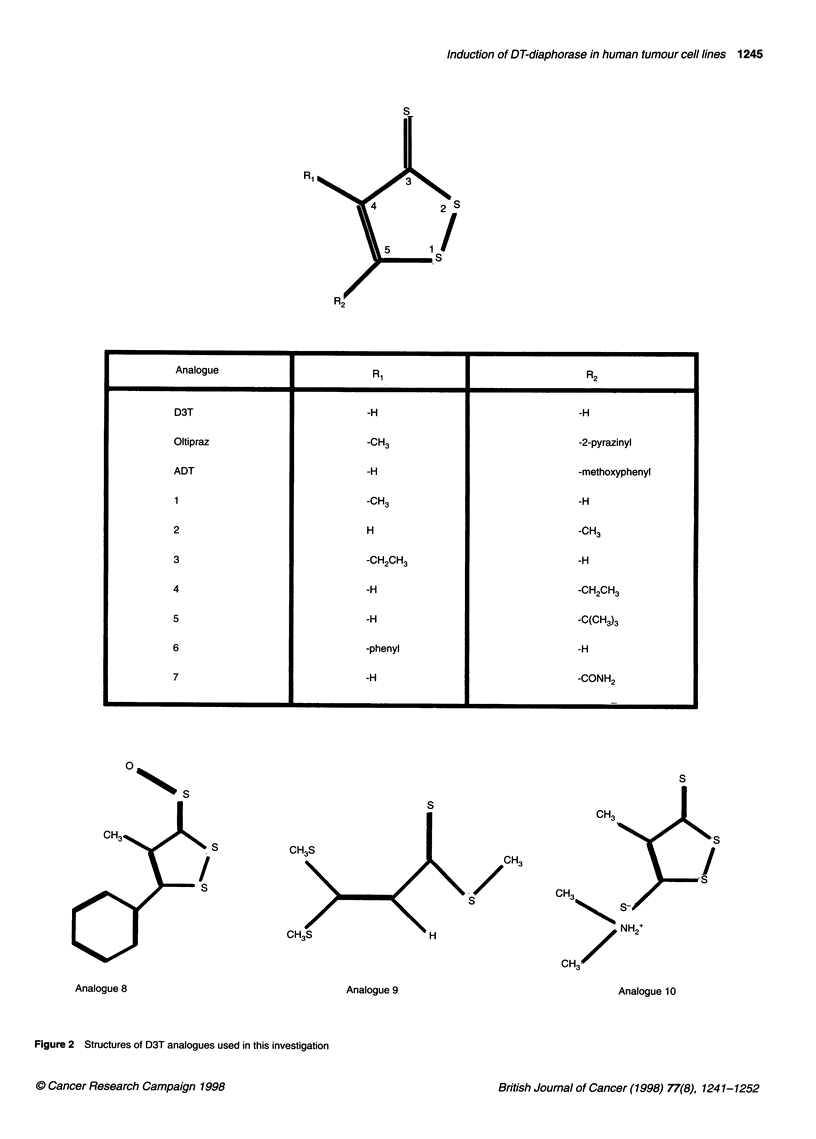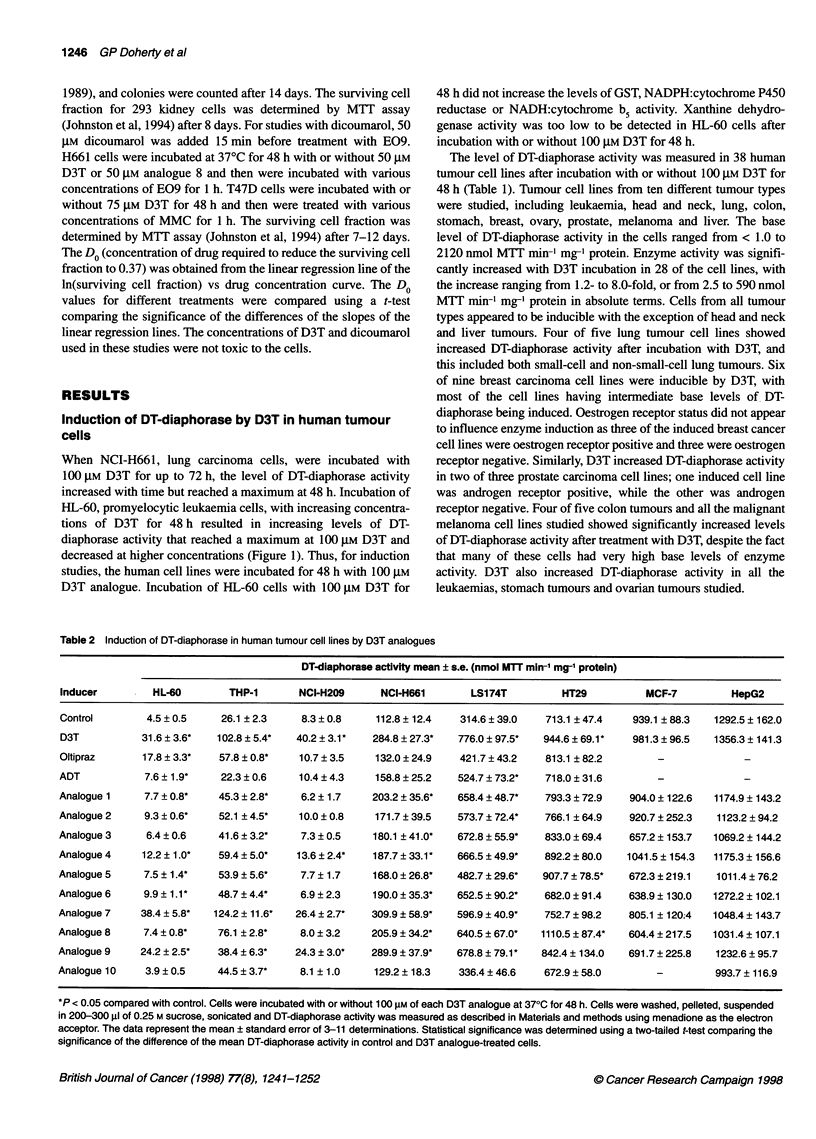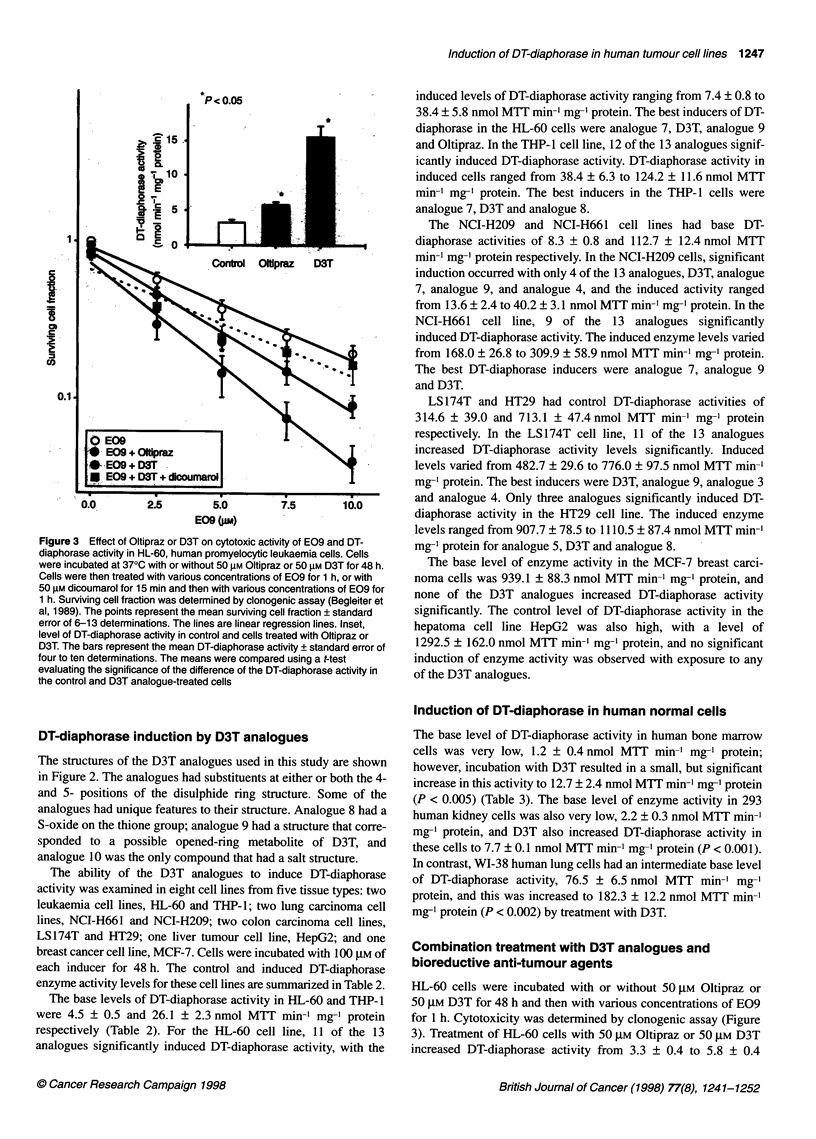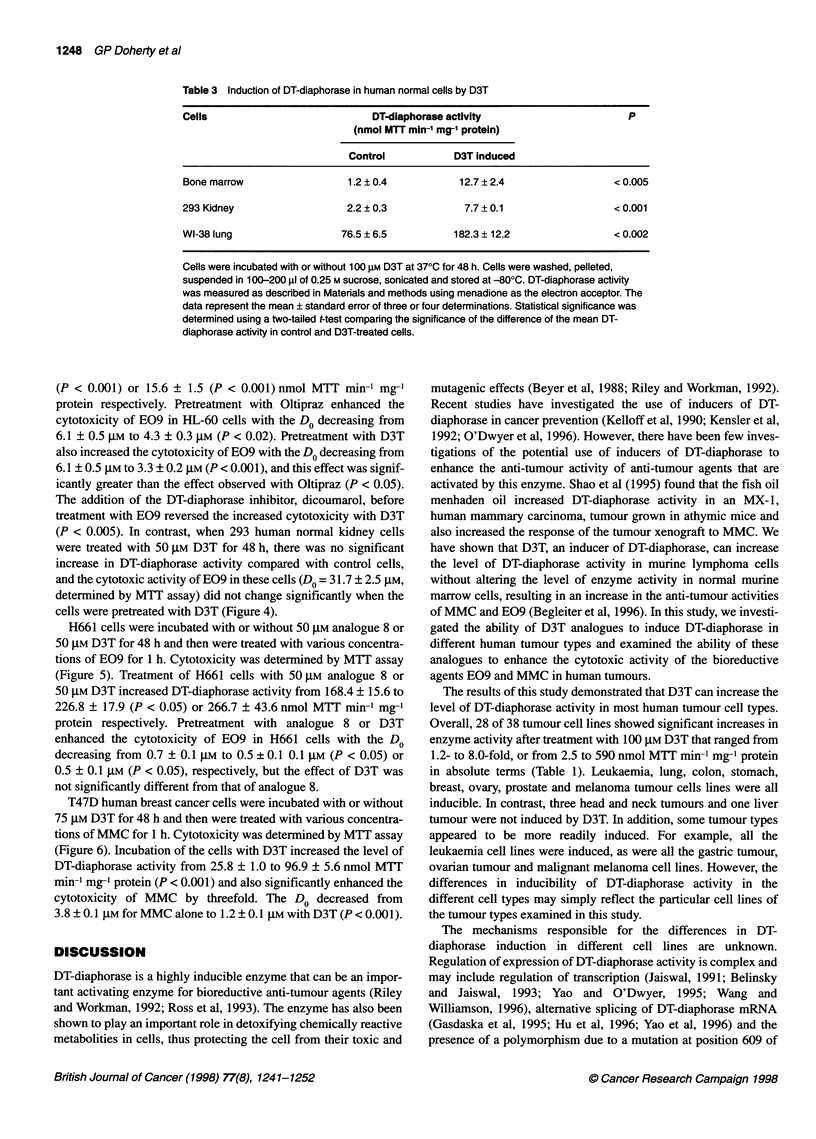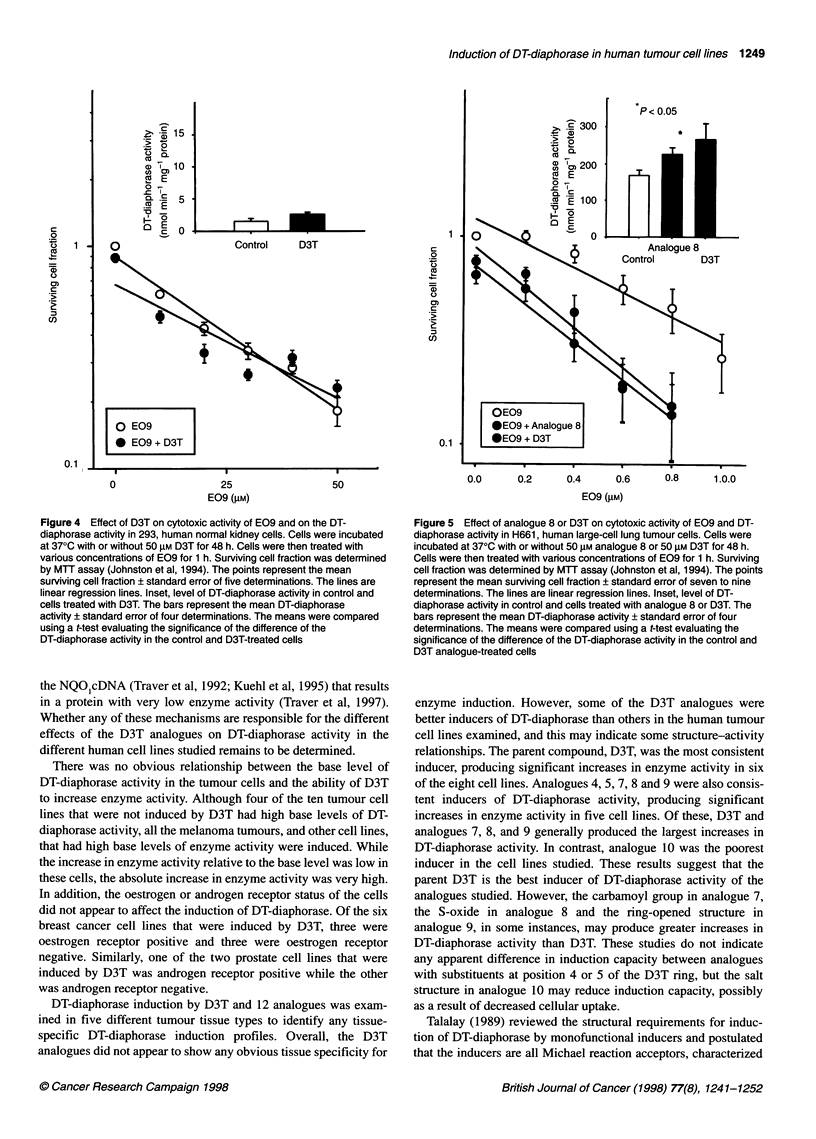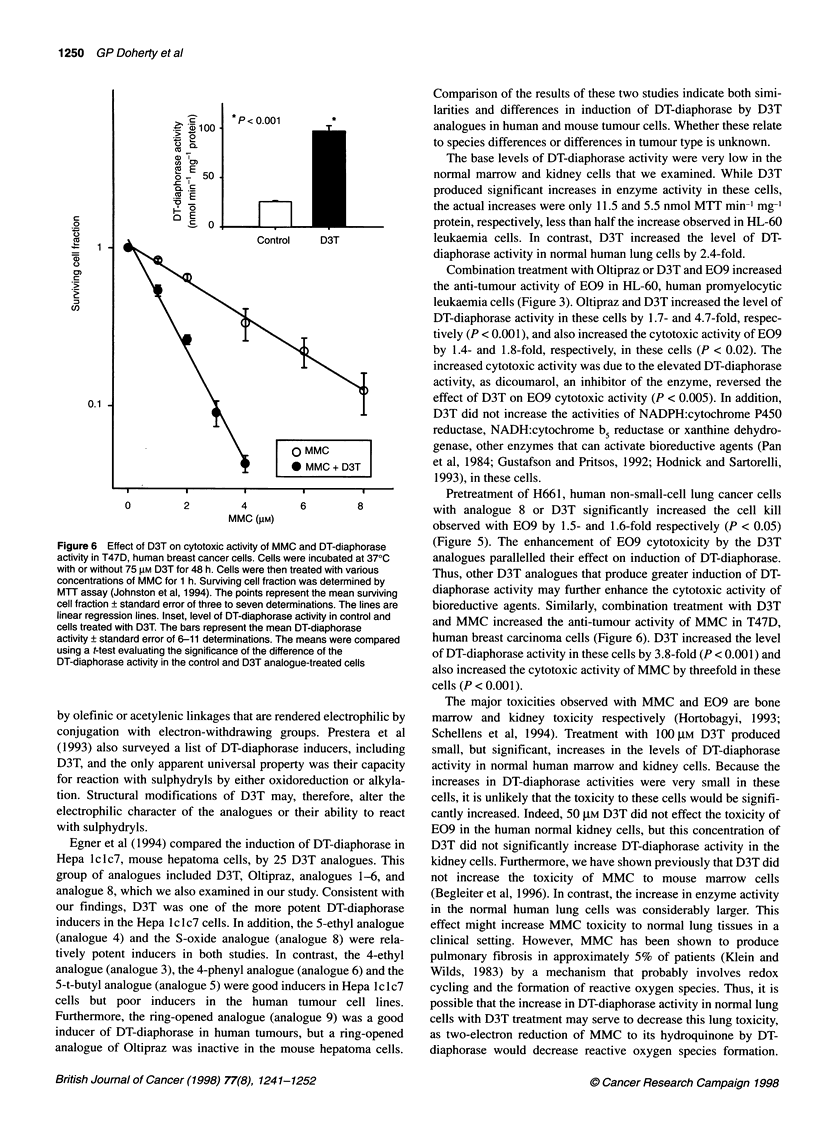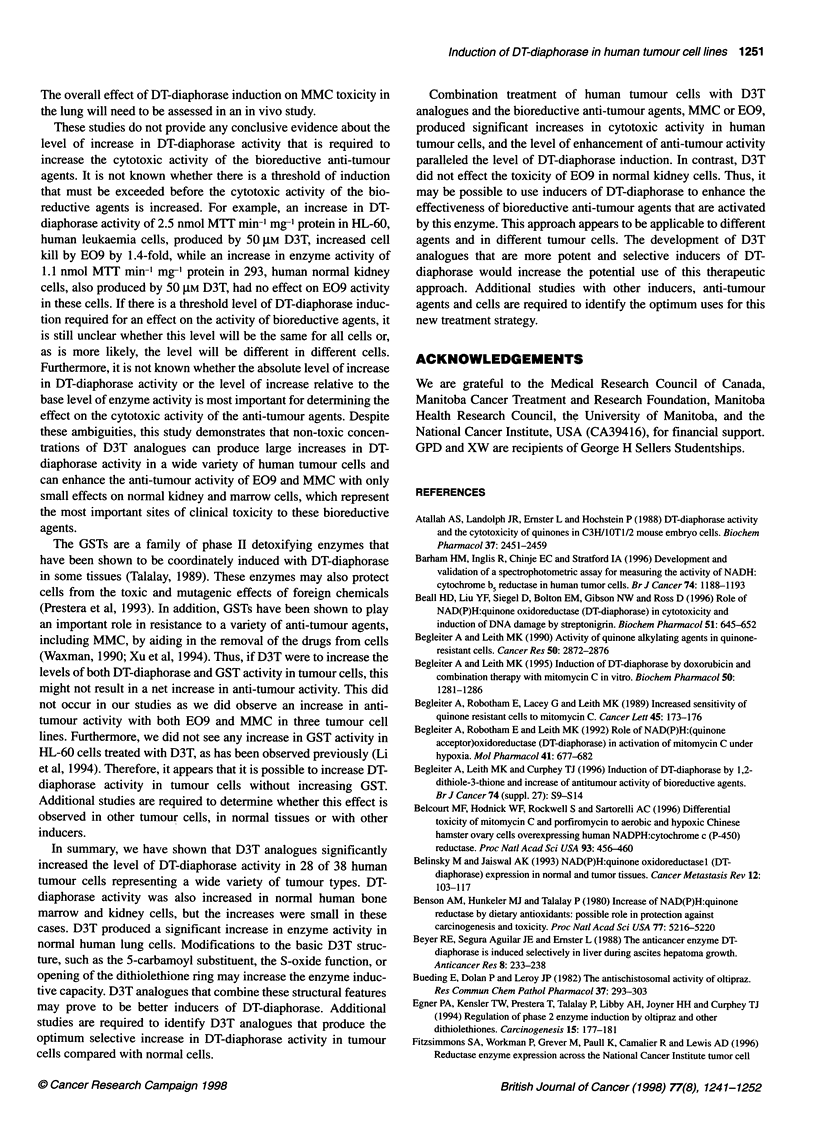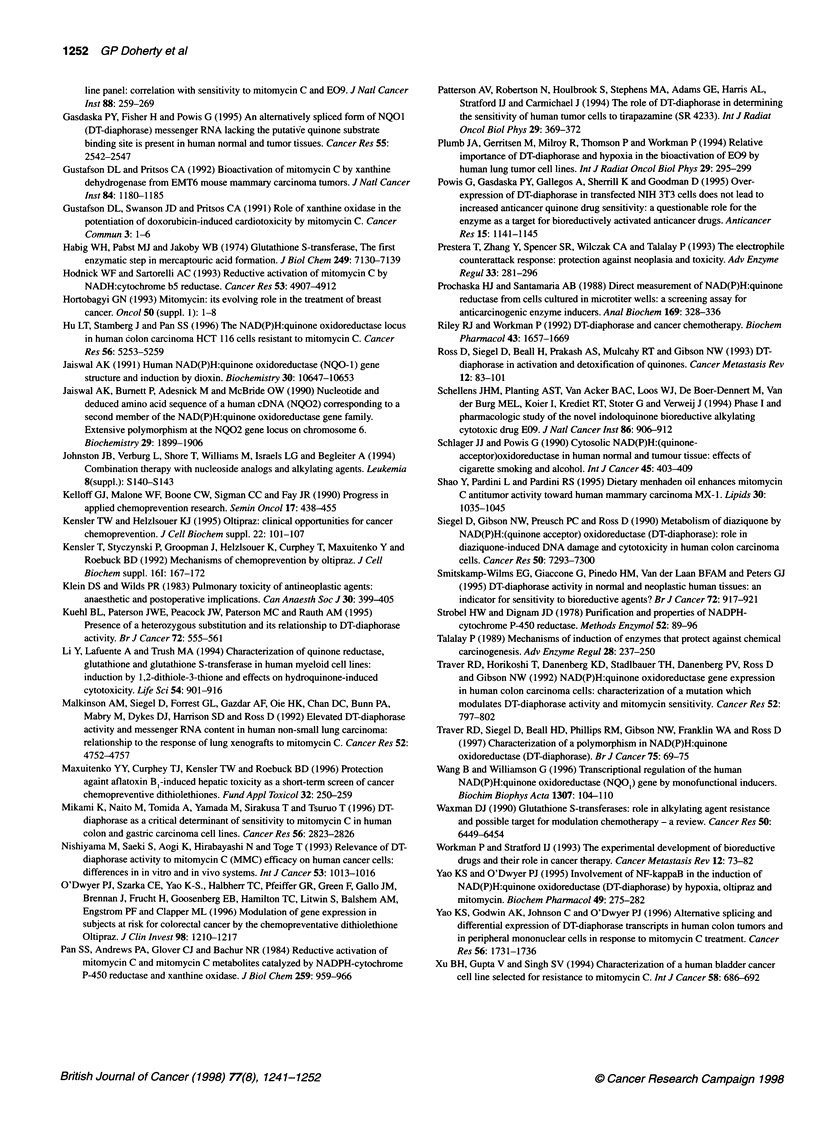# Induction of DT-diaphorase by 1,2-dithiole-3-thiones in human tumour and normal cells and effect on anti-tumour activity of bioreductive agents.

**DOI:** 10.1038/bjc.1998.209

**Published:** 1998-04

**Authors:** G. P. Doherty, M. K. Leith, X. Wang, T. J. Curphey, A. Begleiter

**Affiliations:** Manitoba Institute of Cell Biology, Manitoba Cancer Treatment and Research Foundation, Department of Pharmacology, University of Manitoba, Winnipeg, Canada.

## Abstract

DT-diaphorase is a two-electron-reducing enzyme that is an important activator of bioreductive anti-tumour agents, such as mitomycin C (MMC) and EO9, and is inducible by many compounds, including 1,2-dithiole-3-thiones (D3Ts). We showed previously that D3T selectively increased DT-diaphorase activity in mouse lymphoma cells compared with normal mouse marrow cells, and also increased MMC or EO9 cytotoxic activity in the lymphoma cells with only minor effects in the marrow cells. In this study, we found that D3T significantly increased DT-diaphorase activity in 28 of 38 human tumour cell lines representing ten tissue types with no obvious relationships between the tumour type, or the base level of DT-diaphorase activity, and the ability of D3T to increase the enzyme activity. Induction of DT-diaphorase activity in human tumour cell lines by 12 D3T analogues varied markedly with the D3T structure. D3T also increased DT-diaphorase activity in normal human bone marrow and kidney cells but the increases were small in these cells. In addition, D3T increased the level of enzyme activity in normal human lung cells. Pretreatment of human tumour cells with D3T analogues significantly increased the cytotoxic activity of MMC or EO9 in these cells, and the level of enhancement of anti-tumour activity paralleled the level of DT-diaphorase induction. In contrast, D3T did not effect the toxicity of EO9 in normal kidney cells. These results demonstrate that D3T analogues can increase DT-diaphorase activity in a wide variety of human tumour cells and that this effect can enhance the anti-tumour activity of the bioreductive agents MMC and EO9.


British Journal of Cancer (1998) 77(8), 1241–1252
© 1998 Cancer Research Campaign

### Induction of DT-diaphorase by 1,2-dithiole-3-thiones in human tumour and normal cells and effect on anti-tumour activity of bioreductive agents

**GP Doherty[1,2], MK Leith[1,3], X Wang[1,2], TJ Curphey[4] and A Begleiter[1,2,3]**

[1]Manitoba Institute of Cell Biology, Manitoba Cancer Treatment and Research Foundation, Departments of [2]Pharmacology and Therapeutics and [3]Internal Medicine, University of Manitoba, 100 Olivia Street, Winnipeg, MB, R3E 0V9 Canada; [4]Department of Pathology, Dartmouth College, Hanover, NH, 03755 USA

**Summary** DT-diaphorase is a two-electron-reducing enzyme that is an important activator of bioreductive anti-tumour agents, such as mitomycin C (MMC) and EO9, and is inducible by many compounds, including 1,2-dithiole-3-thiones (D3Ts). We showed previously that D3T selectively increased DT-diaphorase activity in mouse lymphoma cells compared with normal mouse marrow cells, and also increased MMC or EO9 cytotoxic activity in the lymphoma cells with only minor effects in the marrow cells. In this study, we found that D3T significantly increased DT-diaphorase activity in 28 of 38 human tumour cell lines representing ten tissue types with no obvious relationships between the tumour type, or the base level of DT-diaphorase activity, and the ability of D3T to increase the enzyme activity. Induction of DT-diaphorase activity in human tumour cell lines by 12 D3T analogues varied markedly with the D3T structure. D3T also increased DT-diaphorase activity in normal human bone marrow and kidney cells but the increases were small in these cells. In addition, D3T increased the level of enzyme activity in normal human lung cells. Pretreatment of human tumour cells with D3T analogues significantly increased the cytotoxic activity of MMC or EO9 in these cells, and the level of enhancement of anti-tumour activity paralleled the level of DT-diaphorase induction. In contrast, D3T did not effect the toxicity of EO9 in normal kidney cells. These results demonstrate that D3T analogues can increase DT-diaphorase activity in a wide variety of human tumour cells and that this effect can enhance the anti-tumour activity of the bioreductive agents MMC and EO9.

**Keywords:** bioreductive agent; DT-diaphorase; human tumour; 1,2-dithiole-3-thiones; induction

NAD(P)H:(quinone acceptor) oxidoreductase (DT-diaphorase) (EC 1.6.99.2) is a flavoprotein that catalyses obligatory two-electron reduction of quinones, quinone imines, azo dyes and nitrogen oxides (Riley and Workman, 1992). The enzyme is found in all eukaryotes and is present at varying levels in most tissues (Benson et al, 1980; Belinsky and Jaiswal, 1993). DT-diaphorase is located mainly in the cytosol, but 5–10% is membrane bound in mitochondria, microsomes and Golgi apparatus (Riley and Workman, 1992). The enzyme has two identical subunits with individual molecular weights of 32 kDa and requires NADH or NADPH as an electron donor for enzymatic activity (Riley and Workman, 1992). Several DT-diaphorases have been identified in humans (Jaiswal et al, 1990; Jaiswal, 1991), but the $NQO_1$ gene has been most extensively studied and appears to be most important for activation of bioreductive anti-tumour agents (Jaiswal, 1991; Riley and Workman, 1992; Belinsky and Jaiswal, 1993). Enzyme levels have been shown to be relatively high in mouse and/or human stomach, bladder, intestine, colon and kidney, but are usually low in liver, lung and haematopoietic cells (Benson et al, 1980; Schlager and Powis, 1990; Smitskamp-Wilms et al, 1995). DT-diaphorase activity is higher in some tumour cells than in the corresponding normal cells, with elevated levels of enzyme activity having been observed in human liver, colon, breast and lung tumour cells (Schlager and Powis, 1990; Malkinson et al, 1992; Belinsky and Jaiswal, 1993; Smitskamp-Wilms et al, 1995).

DT-diaphorase is a phase II detoxifying enzyme that is involved in metabolizing xenobiotics and carcinogens, thereby protecting the cell from their toxic and mutagenic effects (Beyer et al, 1988; Riley and Workman, 1992). There has been considerable interest in the role of this enzyme, and other phase II enzymes, such as glutathione *S*-transferases (GST), epoxide hydrolase and UDP-glucuronosyltransferases, in early cellular defence mechanisms against tumorigenesis (Beyer et al, 1988; Prestera et al, 1993). DT-diaphorase is induced in many tissues by a wide variety of structurally dissimilar chemicals, including 1,2-dithiole-3-thiones (D3Ts), quinones, diphenols, phenylenediamines, Michael reaction acceptors, isothiocyanates and heavy metals (Prestera et al, 1993). Thus, studies have investigated the use of inducers of DT-diaphorase and other phase II detoxifying enzymes in cancer prevention (Kelloff et al, 1990; Kensler and Helzlsouer, 1995). Oltipraz, a D3T analogue used for treatment of schistosomiasis (Bueding et al, 1982), is an inducer of phase II detoxifying enzymes (Kensler et al, 1992; Egner et al, 1994; Kensler and Helzlsouer, 1995) and inhibits development of tumours in animals (Kelloff et al, 1990; Kensler et al, 1992; Kensler and Helzlsouer, 1995). This agent has low toxicity in animals and is in phase I and II clinical trials as a chemoprotective agent in humans (Kensler et al, 1992; Maxuitenko et al, 1996; O'Dwyer et al, 1996). Because of their ability to induce phase II enzymes and their relatively low toxicity, other D3T analogues are also being investigated as cancer preventative agents (Egner et al, 1994).

DT-diaphorase has been shown to be an important activating enzyme for bioreductive anti-tumour agents, such as mitomycin C (MMC) (Begleiter et al, 1989; Ross et al, 1993; Mikami et al,

*Received 9 June 1997*
*Revised 25 September 1997*
*Accepted 10 October 1997*

*Correspondence to:* A Begleiter

1996), 3-hydroxymethyl-5-aziridinyl-1-methyl-2(1H-indole-4,7-dione)prop-β-en-α-ol (EO9) (Plumb et al, 1994), diaziquone (Siegel et al, 1990), streptonigrin (Beall et al, 1996) and others (Workman and Stratford, 1993) in many systems. Bioreductive agents require intracellular activation by either two-electron-reducing enzymes, such as DT-diaphorase (Ross et al, 1993; Plumb et al, 1994) and xanthine dehydrogenase (EC 1.1.1.204) (Gustafson and Pritsos, 1992) or by one-electron-reducing enzymes, such as NADPH:cytochrome P450 reductase (EC 1.6.2.4) (Pan et al, 1984) and NADH:cytochrome $b_5$ reductase (EC 1.6.2.2) (Hodnick and Sartorelli, 1993). In general, cell lines (Begleiter et al, 1989; Malkinson et al, 1992; Ross et al, 1993; Mikami et al, 1996) or tumour specimens (Nishiyama et al, 1993) with higher levels of DT-diaphorase are more sensitive to MMC. A significant correlation was found between the level of DT-diaphorase activity and sensitivity to MMC and EO9 in the human tumour cell lines of the National Cancer Institute Tumour Cell Line Panel (Fitzsimmons et al, 1996). EO9 activity appears to be particularly sensitive to the level of DT-diaphorase under oxygenated conditions (Plumb et al, 1994). Increasing DT-diaphorase activity in gastric carcinoma (Mikami et al, 1996) or Chinese hamster ovary cells (Belcourt et al, 1996), by transfection of the $NQO_1$ gene, increased the sensitivity of the cells to MMC. In contrast, transfection of the $NQO_1$ gene into NIH 3T3 mouse fibroblasts did not increase MMC activity in these cells (Powis et al, 1995). In addition, DT-diaphorase did not appear to play a major role in activation of MMC in some cell lines in vivo (Nishiyama et al, 1993) or under hypoxia (Begleiter et al, 1992; Belcourt et al, 1996). Furthermore, DT-diaphorase did not effect the toxicity of the benzotriazine di-N-oxide, tirapazamine (Patterson et al, 1994) and protected cells from the toxicity of the quinone agents, menadione (Atallah et al, 1988), hydrolysed benzoquinone mustard, benzoquinone mustard and benzoquinone dimustard (Begleiter and Leith, 1990).

A study by Shao et al (1995) found that high levels of dietary fish oil lowered the growth rate of MX-1, human mammary carcinoma, xenografts in athymic mice and increased DT-diaphorase activity in the tumour. The high levels of fish oil also increased the response of the tumour to MMC. We have previously shown (Begleiter et al, 1996) that D3T selectively increased the level of DT-diaphorase activity in L5178Y murine lymphoma cells with no effect on the activity of this enzyme in normal marrow cells from DBA/2 mice. Combination treatment of L5178Y tumour cells with D3T and MMC produced a twofold increase in cytotoxic activity compared with MMC alone. Similar treatment with D3T and EO9 produced a sevenfold increase in anti-tumour activity compared with EO9 alone. By comparison, D3T did not enhance the activity of MMC in normal mouse marrow cells and produced only a small increase in EO9 cytotoxicity in these cells. The DT-diaphorase inhibitor dicoumarol inhibited the effect of D3T on the anti-tumour activity of the bioreductive agents, supporting the proposal that the enhanced anti-cancer activity was due to the elevated level of enzyme activity.

These findings suggest that inducers of DT-diaphorase, such as D3T analogues, may be useful to enhance the anti-tumour efficacy of bioreductive anti-tumour agents. Although induction of DT-diaphorase by D3T analogues has been studied previously in rodent tissues in the context of cancer chemoprevention, in this study we investigated the ability of D3T analogues to increase the level of DT-diaphorase activity in human tumour and normal cells as a way of enhancing the anti-tumour activity of bioreductive agents. We examined induction of the enzyme in tumour cell lines from different tissues and in cell lines having different base levels of DT-diaphorase. It has been shown previously (Egner et al, 1994) that induction of DT-diaphorase in mouse hepatoma cells was dependent on the chemical structure of the D3T analogue. Thus, we also investigated the ability of D3T analogues to induce DT-diaphorase activity in human tumour cell lines to identify structure–activity relationships that might be useful for developing more potent and selective enzyme inducers. Finally, we evaluated the effect of combining D3T analogues with MMC or EO9 on the cytotoxic activity of these bioreductive anti-tumour agents in human tumour and normal cell lines.

### MATERIALS AND METHODS

### Materials

All media, fetal bovine serum (FBS) and insulin transferrin selenium (ITS) were obtained from Gibco BRL (Grand Island, NY, USA). All reagents for the DT-diaphorase assay, hydrocortisone, Ficoll 400, lactalbumin hydrosylate, 3-[4,5-dimethylthiazol-2-yl]-2,5-diphenyltetrazolium bromide (MTT) and MMC were from Sigma (St Louis, MO, USA). EO9 was kindly supplied by Dr HR Hendriks, New Drug Development Office, European Organization for Research and Treatment of Cancer, Amsterdam, The Netherlands. MMC was dissolved in phosphate-buffered saline (PBS), while EO9 was dissolved either in dimethyl sulphoxide (DMSO) or DMSO–ethanol (2:3, v:v). The final concentration of DMSO or ethanol did not exceed 1%. The D3T analogues were synthesized by Dr TJ Curphey. D3T analogues were prepared in ethanol at a concentration of $2 \times 10^{-2}$ M with the exceptions of ADT, which was prepared at $1 \times 10^{-2}$ M in ethanol, and Oltipraz, which was prepared at $2 \times 10^{-2}$ M in DMSO or at $5 \times 10^{-3}$ M in acetone. The final concentration of acetone did not exceed 2% and that of DMSO or ethanol did not exceed 1% in the cell incubation media. Appropriate media and FBS at pH 7.2 were used for all DT-diaphorase induction studies and incubations with MMC or EO9.

### Cells

HL-60, promyelocytic leukaemia cells, were obtained from Dr AH Greenberg, Manitoba Institute of Cell Biology, Winnipeg, Canada, and were grown in RPMI 1640 medium and 10% FBS. THP-1, monocytic leukaemia cells, were obtained from Dr D Houston, Manitoba Institute of Cell Biology, and were grown in RPMI 1640 and 10% FBS. WIL-2, B lymphoblastoid leukaemia cells, were purchased from American Type Culture Collection (Rockville, MD) and were grown in RPMI 1640 and 10% FBS. SCC-25, tongue squamous carcinoma cells, were from American Type Culture Collection and were grown in Dulbecco's Modified Eagle Medium (DMEM) F-12(1:1) with 0.4 μg $ml^{-1}$ of hydrocortisone and 10% FBS. Detroit 562, pharynx carcinoma cells, were from American Type Culture Collection and were grown in DMEM/F-12(1:1) with 0.1% lactalbumin hydrosylate and 10% FBS. FaDu, pharynx squamous carcinoma cells, were from American Type Culture Collection and were grown in DMEM/F-12(1:1) and 10% FBS. NCI-H596, lung adenosquamous carcinoma cells, NCI-H209, lung small-cell carcinoma cells, NCI-H661, lung large-cell carcinoma cells, and NCI-H520, lung squamous carcinoma cells, were obtained from American Type Culture Collection and were grown in RPMI 1640 and 10% FBS. NCI-H125, lung non-small-cell adenocarcinoma cells, were obtained from Dr SS Pan, University

© Cancer Research Campaign 1998

**Figure 1** Induction of DT-diaphorase in HL-60 cells by D3T. HL-60 cells were incubated with various concentrations of D3T at 37°C for 48 h and DT-diaphorase activity was measured as described in Materials and methods. The data represent the mean ± standard error of four or five determinations of Maryland Cancer Center, Baltimore, MD, USA, and were grown in RPMI 1640 and 10% FBS. Colo320DM, colon carcinoma cells, were from American Type Culture Collection and were grown in RPMI 1640 and 10% FBS. HCT116 and LS174T, colon carcinoma cells, were from American Type Culture Collection and were grown in DMEM/F-12(1:1) and 10% FBS. Colo205, colon adenocarcinoma cells, were obtained from Dr AH Greenberg and were grown in RPMI 1640 and 10% FBS, while HT29, colon adenocarcinoma cells, were obtained from Dr JB Johnston, Manitoba Cancer Treatment and Research Foundation, Winnipeg, Canada, and were grown in RPMI 1640 and 10% FBS. RF-48 and RF-1, gastric adenocarcinoma cells, were from Dr JA Wright, Manitoba Institute of Cell Biology, and were grown in alpha minimal essential medium (MEM) and 10% FBS. AGS, gastric adenocarcinoma cells, and Kato III, gastric carcinoma cells, were obtained from Dr JA Wright and were grown in RPMI 1640 and 10% FBS. MDA-MB-231, breast adenocarcinoma cells, were from American Type Culture Collection and were grown in IMDM and 10% FBS. MDA-MB-468, breast adenocarcinoma cells, were obtained from American Type Culture Collection and were grown in DMEM/F-12(1:1) with 1% ITS and 10% FBS. T47D, breast ductal carcinoma cells, were from Dr S Mai, Manitoba Institute of Cell Biology, and were grown in RPMI 1640 with 1% ITS and 10% FBS. BT474, breast ductal carcinoma cells, were from Dr SS Pan and were grown in DMEM/F-12(1:1) with 1% ITS and 10% FBS. SK-Br-3, breast adenocarcinoma cells, were obtained from American Type Culture Collection and were grown in McCoy's medium and 10% FBS. MDA-MB-435, breast ductal carcinoma cells, were from Dr EA Turley, Manitoba Institute of Cell Biology, and were grown in DMEM/F-12(1:1) and 10% FBS. HS578T, breast ductal carcinoma cells, and ZR-75-1, breast carcinoma cells, were obtained from Dr SS Pan and were grown in DMEM/F-12(1:1) and 10% FBS. MCF-7, breast adenocarcinoma cells, were from Dr AH Greenberg and were grown in RPMI 1640 and 10% FBS. OVCAR-3, ovarian adenocarcinoma cells, were from American Type Culture Collection and were grown in RPMI 1640 with 1% ITS and 20% FBS. SK-OV-3, ovarian adenocarcinoma cells, were from American Type Culture Collection and were grown in McCoy's medium and 10% FBS. PC-3, prostate adenocarcinoma cells, and DU145, prostate carcinoma cells, were obtained from Dr J Dodd, University of Manitoba, Winnipeg, Canada, and were grown in DMEM/F-12(1:1) and 10% FBS. LnCAP, prostate adenocarcinoma cells, were from Dr J Dodd and were grown in RPMI 1640 and 10% FBS. SK-MEL-28, SK-MEL-2 and SK-MEL-5, malignant melanoma cells, were obtained from American Type Culture Collection and were grown in DMEM/F-12(1:1) and 10% FBS. HepG2, hepatocellular carcinoma cells, were from American Type Culture Collection and were grown in alpha MEM and 15% FBS. Normal marrow specimens were obtained from marrow donated for transplantation and mononuclear cells were isolated using a Ficoll-Hypaque gradient (Johnston et al, 1994). WI-38, human embryonic lung cells, were obtained from Dr JA Wright and were grown in alpha MEM and 10% FBS, while 293, human embryonic kidney cells, were from Dr M Mowat, Manitoba Institute of Cell Biology, and were grown in DMEM/F-12(1:1) and 10% FBS.

### Induction of DT-diaphorase

Exponentially growing cells were treated with D3T analogues at 37°C for various periods of time. Cells were washed, pelleted, suspended in 100–200 μl of 0.25 M sucrose, sonicated and stored at −80°C. DT-diaphorase activity was measured, using menadione as the electron acceptor, by the assay of Prochaska and Santamaria (1988), modified for use in a semi-microcuvette. Briefly, 5–100 μg of protein was assayed for DT-diaphorase activity by adding the appropriate volume of sucrose sonicate to a semi-microcuvette containing 750 μl of the assay stock solution (Prochaska and Santamaria, 1988). The increase in absorbance at 25°C at 610 nm was followed for up to 10 min using a Cary I spectrophotometer. Duplicate cuvettes were prepared for each sample, one for total activity and one with 10 μM dicoumarol. The DT-diaphorase activity reported was the dicoumarol-inhibitable activity. DT-diaphorase activity was expressed as nmol MTT min$^{-1}$ mg$^{-1}$ protein. Enzyme activities as low as 1.0 nmol MTT min$^{-1}$ mg$^{-1}$ protein could be detected with this assay procedure. Protein concentration was measured using the Bio-Rad DC Kit (Bio-Rad, Mississauga, Canada) with gamma globulin as standard. The concentrations of the D3T analogues used in these studies produced little or no toxicity to the cells during the incubation period.

### Measurement of GST activity

GST activity was measured in the supernatant of cell sonicates by a previously described spectrophotometric procedure (Habig et al, 1974) using 1-chloro-2,4-dinitrobenzene as substrate.

### Measurement of NADPH:cytochrome P450 reductase activity

NADPH-cytochrome P450 reductase activity was determined in supernatants from cell sonicates by a spectrophotometric assay using cytochrome c as the artificial electron acceptor (Strobel and Digman, 1978).

### Measurement of NADH:cytochrome $b_5$ reductase activity

NADH:cytochrome $b_5$ reductase activity was determined spectrophotometrically using the method of Barham et al (1996), which measures the pHMB-inhibitable, NADH-dependent reduction of cytochrome c.

**Table 1** Induction of DT-diaphorase in human tumour cell lines by D3T

| Tumour type | Cell line | DT-diaphorase activity Mean ± s.e. (nmol MTT min$^{-1}$ mg$^{-1}$ protein) | | *P* |
|---|---|---|---|---|
| | | Control | D3T induced | |
| Leukaemia | HL-60 | 4.0 ± 0.5 | 31.1 ± 3.4 | <0.001 |
| | THP-1 | 26.1 ± 2.3 | 102.8 ± 5.4 | <0.001 |
| | WIL-2 | 58.8 ± 4.4 | 139.2 ± 8.9 | <0.001 |
| Head and neck | SCC-25 | 39.7 ± 7.8 | 66.1 ± 16.7 | NS |
| | Detroit 562 | 167.7 ± 27.9 | 199.7 ± 39.3 | NS |
| | FaDu | 463.2 ± 29.9 | 444.1 ± 45.2 | NS |
| Lung | NCI-H596 | 1.0 ± 0.2 | 3.5 ± 0.6 | <0.005 |
| | NCI-H209 | 8.3 ± 0.8 | 40.2 ± 3.1 | <0.001 |
| | NCI-H661 | 112.8 ± 12.4 | 284.8 ± 27.3 | <0.001 |
| | NCI-H520 | 230.1 ± 31.3 | 267.0 ± 36.5 | NS |
| | NCI-H125 | 689.5 ± 39.0 | 1136.0 ± 52.1 | <0.001 |
| Colon | Colo320DM | ND | ND | – |
| | HCT116 | 89.8 ± 11.9 | 205.1 ± 35.3 | <0.02 |
| | LS174T | 314.6 ± 39.0 | 776.1 ± 97.5 | <0.005 |
| | Colo205 | 671.2 ± 64.5 | 1117.3 ± 111.5 | <0.005 |
| | HT29 | 713.1 ± 47.4 | 944.6 ± 69.1 | <0.02 |
| Stomach | RF-48 | 6.9 ± 0.3 | 22.7 ± 1.0 | <0.001 |
| | RF-1 | 7.7 ± 0.5 | 22.7 ± 0.4 | <0.001 |
| | AGS | 138.5 ± 25.7 | 294.6 ± 31.9 | <0.005 |
| | Kato III | 167.0 ± 6.7 | 289.3 ± 11.0 | <0.001 |
| Breast | MDA-MB-231 | ND | 1.9 ± 0.5 | – |
| | MDA-MB-468 | 4.4 ± 2.5 | ND | – |
| | T47D | 27.9 ± 1.2 | 97.6 ± 4.5 | <0.001 |
| | BT474 | 191.5 ± 10.1 | 430.8 ± 36.1 | <0.001 |
| | SK-Br-3 | 213.0 ± 1.5 | 331.0 ± 13.7 | <0.002 |
| | MDA-MB-435 | 232.2 ± 16.2 | 291.7 ± 16.0 | <0.02 |
| | HS578T | 237.9 ± 13.9 | 420.8 ± 19.0 | <0.001 |
| | ZR-75-1 | 355.5 ± 43.0 | 592.5 ± 71.7 | <0.02 |
| | MCF-7 | 939.1 ± 88.3 | 981.2 ± 96.5 | NS |
| Ovary | OVCAR-3 | 47.2 ± 5.9 | 129.3 ± 10.9 | <0.001 |
| | SK-OV-3 | 177.8 ± 8.6 | 259.0 ± 15.9 | <0.002 |
| Prostate | PC-3 | 149.3 ± 5.0 | 183.0 ± 1.7 | <0.005 |
| | LnCAP | 166.0 ± 15.1 | 266.2 ± 34.5 | <0.02 |
| | DU145 | 652.3 ± 30.0 | 632.0 ± 20.1 | NS |
| Skin | SK-MEL-28 | 586.7 ± 19.6 | 828.7 ± 38.4 | <0.005 |
| | SK-MEL-2 | 666.5 ± 30.6 | 796.3 ± 36.5 | <0.05 |
| | SK-MEL-5 | 2120.0 ± 51.3 | 2710.0 ± 60.8 | <0.002 |
| Liver | HepG2 | 1292.5 ± 162.0 | 1356.3 ± 141.3 | NS |

ND, not detected; NS, not significant. Cells were incubated with or without 100 μM D3T at 37°C for 48 h. Cells were washed, pelleted, suspended in 100–200 μl of 0.25 M sucrose, sonicated and stored at –80°C. DT-diaphorase activity was measured as described in Materials and methods using menadione as the electron acceptor. The data represent the mean ± standard error of 3–15 determinations. Statistical significance was determined using a two-tailed *t*-test comparing the significance of the difference of the mean DT-diaphorase activity in control and D3T-treated cells.

### Measurement of xanthine dehydrogenase activity

Xanthine dehydrogenase activity was determined using the spectrophotometric method of Gustafson et al (1991). This method follows the formation of uric acid from xanthine in the presence and absence of $NAD^+$ to distinguish the xanthine dehydrogenase and xanthine oxygenase forms of the enzyme.

### Cytotoxicity studies

HL-60, human promyelocytic leukaemia cells, or 293, human normal kidney cells, were incubated with or without 50 μM D3T or 50 μM Oltipraz for 48 h and then were treated with various concentrations of EO9 for 1 h. The surviving cell fraction for HL-60 cells was determined by clonogenic assay (Begleiter et al,

 © Cancer Research Campaign 1998

| Analogue | $R_1$ | $R_2$ |
|---|---|---|
| D3T | -H | -H |
| Oltipraz | $-CH_3$ | -2-pyrazinyl |
| ADT | -H | -methoxyphenyl |
| 1 | $-CH_3$ | -H |
| 2 | H | $-CH_3$ |
| 3 | $-CH_2CH_3$ | -H |
| 4 | -H | $-CH_2CH_3$ |
| 5 | -H | $-C(CH_3)_3$ |
| 6 | -phenyl | -H |
| 7 | -H | $-CONH_2$ |

Analogue 8

Analogue 9

Analogue 10

**Figure 2** Structures of D3T analogues used in this investigation

1989), and colonies were counted after 14 days. The surviving cell fraction for 293 kidney cells was determined by MTT assay (Johnston et al, 1994) after 8 days. For studies with dicoumarol, 50 μM dicoumarol was added 15 min before treatment with EO9. H661 cells were incubated at 37°C for 48 h with or without 50 μM D3T or 50 μM analogue 8 and then were incubated with various concentrations of EO9 for 1 h. T47D cells were incubated with or without 75 μM D3T for 48 h and then were treated with various concentrations of MMC for 1 h. The surviving cell fraction was determined by MTT assay (Johnston et al, 1994) after 7–12 days. The $D_0$ (concentration of drug required to reduce the surviving cell fraction to 0.37) was obtained from the linear regression line of the ln(surviving cell fraction) vs drug concentration curve. The $D_0$ values for different treatments were compared using a *t*-test comparing the significance of the differences of the slopes of the linear regression lines. The concentrations of D3T and dicoumarol used in these studies were not toxic to the cells.

### RESULTS

### Induction of DT-diaphorase by D3T in human tumour cells

When NCI-H661, lung carcinoma cells, were incubated with 100 μM D3T for up to 72 h, the level of DT-diaphorase activity increased with time but reached a maximum at 48 h. Incubation of HL-60, promyelocytic leukaemia cells, with increasing concentrations of D3T for 48 h resulted in increasing levels of DT-diaphorase activity that reached a maximum at 100 μM D3T and decreased at higher concentrations (Figure 1). Thus, for induction studies, the human cell lines were incubated for 48 h with 100 μM D3T analogue. Incubation of HL-60 cells with 100 μM D3T for 48 h did not increase the levels of GST, NADPH:cytochrome P450 reductase or NADH:cytochrome $b_5$ activity. Xanthine dehydrogenase activity was too low to be detected in HL-60 cells after incubation with or without 100 μM D3T for 48 h.

The level of DT-diaphorase activity was measured in 38 human tumour cell lines after incubation with or without 100 μM D3T for 48 h (Table 1). Tumour cell lines from ten different tumour types were studied, including leukaemia, head and neck, lung, colon, stomach, breast, ovary, prostate, melanoma and liver. The base level of DT-diaphorase activity in the cells ranged from < 1.0 to 2120 nmol MTT $min^{-1}$ $mg^{-1}$ protein. Enzyme activity was significantly increased with D3T incubation in 28 of the cell lines, with the increase ranging from 1.2- to 8.0-fold, or from 2.5 to 590 nmol MTT $min^{-1}$ $mg^{-1}$ protein in absolute terms. Cells from all tumour types appeared to be inducible with the exception of head and neck and liver tumours. Four of five lung tumour cell lines showed increased DT-diaphorase activity after incubation with D3T, and this included both small-cell and non-small-cell lung tumours. Six of nine breast carcinoma cell lines were inducible by D3T, with most of the cell lines having intermediate base levels of DT-diaphorase being induced. Oestrogen receptor status did not appear to influence enzyme induction as three of the induced breast cancer cell lines were oestrogen receptor positive and three were oestrogen receptor negative. Similarly, D3T increased DT-diaphorase activity in two of three prostate carcinoma cell lines; one induced cell line was androgen receptor positive, while the other was androgen receptor negative. Four of five colon tumours and all the malignant melanoma cell lines studied showed significantly increased levels of DT-diaphorase activity after treatment with D3T, despite the fact that many of these cells had very high base levels of enzyme activity. D3T also increased DT-diaphorase activity in all the leukaemias, stomach tumours and ovarian tumours studied.

**Table 2** Induction of DT-diaphorase in human tumour cell lines by D3T analogues

| | DT-diaphorase activity mean ± s.e. (nmol MTT $min^{-1}$ $mg^{-1}$ protein) | | | | | | | |
|---|---|---|---|---|---|---|---|---|
| Inducer | HL-60 | THP-1 | NCI-H209 | NCI-H661 | LS174T | HT29 | MCF-7 | HepG2 |
| Control | 4.5 ± 0.5 | 26.1 ± 2.3 | 8.3 ± 0.8 | 112.8 ± 12.4 | 314.6 ± 39.0 | 713.1 ± 47.4 | 939.1 ± 88.3 | 1292.5 ± 162.0 |
| D3T | 31.6 ± 3.6* | 102.8 ± 5.4* | 40.2 ± 3.1* | 284.8 ± 27.3* | 776.0 ± 97.5* | 944.6 ± 69.1* | 981.3 ± 96.5 | 1356.3 ± 141.3 |
| Oltipraz | 17.8 ± 3.3* | 57.8 ± 0.8* | 10.7 ± 3.5 | 132.0 ± 24.9 | 421.7 ± 43.2 | 813.1 ± 82.2 | – | – |
| ADT | 7.6 ± 1.9* | 22.3 ± 0.6 | 10.4 ± 4.3 | 158.8 ± 25.2 | 524.7 ± 73.2* | 718.0 ± 31.6 | – | – |
| Analogue 1 | 7.7 ± 0.8* | 45.3 ± 2.8* | 6.2 ± 1.7 | 203.2 ± 35.6* | 658.4 ± 48.7* | 793.3 ± 72.9 | 904.0 ± 122.6 | 1174.9 ± 143.2 |
| Analogue 2 | 9.3 ± 0.6* | 52.1 ± 4.5* | 10.0 ± 0.8 | 171.7 ± 39.5 | 573.7 ± 72.4* | 766.1 ± 64.9 | 920.7 ± 252.3 | 1123.2 ± 94.2 |
| Analogue 3 | 6.4 ± 0.6 | 41.6 ± 3.2* | 7.3 ± 0.5 | 180.1 ± 41.0* | 672.8 ± 55.9* | 833.0 ± 69.4 | 657.2 ± 153.7 | 1069.2 ± 144.2 |
| Analogue 4 | 12.2 ± 1.0* | 59.4 ± 5.0* | 13.6 ± 2.4* | 187.7 ± 33.1* | 666.5 ± 49.9* | 892.2 ± 80.0 | 1041.5 ± 154.3 | 1175.3 ± 156.6 |
| Analogue 5 | 7.5 ± 1.4* | 53.9 ± 5.6* | 7.7 ± 1.7 | 168.0 ± 26.8* | 482.7 ± 29.6* | 907.7 ± 78.5* | 672.3 ± 219.1 | 1011.4 ± 76.2 |
| Analogue 6 | 9.9 ± 1.1* | 48.7 ± 4.4* | 6.9 ± 2.3 | 190.0 ± 35.3* | 652.5 ± 90.2* | 682.0 ± 91.4 | 638.9 ± 130.0 | 1272.2 ± 102.1 |
| Analogue 7 | 38.4 ± 5.8* | 124.2 ± 11.6* | 26.4 ± 2.7* | 309.9 ± 58.9* | 596.9 ± 40.9* | 752.7 ± 98.2 | 805.1 ± 120.4 | 1048.4 ± 143.7 |
| Analogue 8 | 7.4 ± 0.8* | 76.1 ± 2.8* | 8.0 ± 3.2 | 205.9 ± 34.2* | 640.5 ± 67.0* | 1110.5 ± 87.4* | 604.4 ± 217.5 | 1031.4 ± 107.1 |
| Analogue 9 | 24.2 ± 2.5* | 38.4 ± 6.3* | 24.3 ± 3.0* | 289.9 ± 37.9* | 678.8 ± 79.1* | 842.4 ± 134.0 | 691.7 ± 225.8 | 1232.6 ± 95.7 |
| Analogue 10 | 3.9 ± 0.5 | 44.5 ± 3.7* | 8.1 ± 1.0 | 129.2 ± 18.3 | 336.4 ± 46.6 | 672.9 ± 58.0 | – | 993.7 ± 116.9 |

*$P < 0.05$ compared with control. Cells were incubated with or without 100 μM of each D3T analogue at 37°C for 48 h. Cells were washed, pelleted, suspended in 200–300 μl of 0.25 M sucrose, sonicated and DT-diaphorase activity was measured as described in Materials and methods using menadione as the electron acceptor. The data represent the mean ± standard error of 3–11 determinations. Statistical significance was determined using a two-tailed *t*-test comparing the significance of the difference of the mean DT-diaphorase activity in control and D3T analogue-treated cells.

 © Cancer Research Campaign 1998

**Figure 3** Effect of Oltipraz or D3T on cytotoxic activity of EO9 and DT-diaphorase activity in HL-60, human promyelocytic leukaemia cells. Cells were incubated at 37°C with or without 50 μM Oltipraz or 50 μM D3T for 48 h. Cells were then treated with various concentrations of EO9 for 1 h, or with 50 μM dicoumarol for 15 min and then with various concentrations of EO9 for 1 h. Surviving cell fraction was determined by clonogenic assay (Begleiter et al, 1989). The points represent the mean surviving cell fraction ± standard error of 6–13 determinations. The lines are linear regression lines. Inset, level of DT-diaphorase activity in control and cells treated with Oltipraz or D3T. The bars represent the mean DT-diaphorase activity ± standard error of four to ten determinations. The means were compared using a *t*-test evaluating the significance of the difference of the DT-diaphorase activity in the control and D3T analogue-treated cells

### DT-diaphorase induction by D3T analogues

The structures of the D3T analogues used in this study are shown in Figure 2. The analogues had substituents at either or both the 4- and 5- positions of the disulphide ring structure. Some of the analogues had unique features to their structure. Analogue 8 had a S-oxide on the thione group; analogue 9 had a structure that corresponded to a possible opened-ring metabolite of D3T, and analogue 10 was the only compound that had a salt structure.

The ability of the D3T analogues to induce DT-diaphorase activity was examined in eight cell lines from five tissue types: two leukaemia cell lines, HL-60 and THP-1; two lung carcinoma cell lines, NCI-H661 and NCI-H209; two colon carcinoma cell lines, LS174T and HT29; one liver tumour cell line, HepG2; and one breast cancer cell line, MCF-7. Cells were incubated with 100 μM of each inducer for 48 h. The control and induced DT-diaphorase enzyme activity levels for these cell lines are summarized in Table 2.

The base levels of DT-diaphorase activity in HL-60 and THP-1 were 4.5 ± 0.5 and 26.1 ± 2.3 nmol MTT $min^{-1}$ $mg^{-1}$ protein respectively (Table 2). For the HL-60 cell line, 11 of the 13 analogues significantly induced DT-diaphorase activity, with the induced levels of DT-diaphorase activity ranging from 7.4 ± 0.8 to 38.4 ± 5.8 nmol MTT $min^{-1}$ $mg^{-1}$ protein. The best inducers of DT-diaphorase in the HL-60 cells were analogue 7, D3T, analogue 9 and Oltipraz. In the THP-1 cell line, 12 of the 13 analogues significantly induced DT-diaphorase activity. DT-diaphorase activity in induced cells ranged from 38.4 ± 6.3 to 124.2 ± 11.6 nmol MTT $min^{-1}$ $mg^{-1}$ protein. The best inducers in the THP-1 cells were analogue 7, D3T and analogue 8.

The NCI-H209 and NCI-H661 cell lines had base DT-diaphorase activities of 8.3 ± 0.8 and 112.7 ± 12.4 nmol MTT $min^{-1}$ $mg^{-1}$ protein respectively. In the NCI-H209 cells, significant induction occurred with only 4 of the 13 analogues, D3T, analogue 7, analogue 9, and analogue 4, and the induced activity ranged from 13.6 ± 2.4 to 40.2 ± 3.1 nmol MTT $min^{-1}$ $mg^{-1}$ protein. In the NCI-H661 cell line, 9 of the 13 analogues significantly induced DT-diaphorase activity. The induced enzyme levels varied from 168.0 ± 26.8 to 309.9 ± 58.9 nmol MTT $min^{-1}$ $mg^{-1}$ protein. The best DT-diaphorase inducers were analogue 7, analogue 9 and D3T.

LS174T and HT29 had control DT-diaphorase activities of 314.6 ± 39.0 and 713.1 ± 47.4 nmol MTT $min^{-1}$ $mg^{-1}$ protein respectively. In the LS174T cell line, 11 of the 13 analogues increased DT-diaphorase activity levels significantly. Induced levels varied from 482.7 ± 29.6 to 776.0 ± 97.5 nmol MTT $min^{-1}$ $mg^{-1}$ protein. The best inducers were D3T, analogue 9, analogue 3 and analogue 4. Only three analogues significantly induced DT-diaphorase activity in the HT29 cell line. The induced enzyme levels ranged from 907.7 ± 78.5 to 1110.5 ± 87.4 nmol MTT $min^{-1}$ $mg^{-1}$ protein for analogue 5, D3T and analogue 8.

The base level of enzyme activity in the MCF-7 breast carcinoma cells was 939.1 ± 88.3 nmol MTT $min^{-1}$ $mg^{-1}$ protein, and none of the D3T analogues increased DT-diaphorase activity significantly. The control level of DT-diaphorase activity in the hepatoma cell line HepG2 was also high, with a level of 1292.5 ± 162.0 nmol MTT $min^{-1}$ $mg^{-1}$ protein, and no significant induction of enzyme activity was observed with exposure to any of the D3T analogues.

### Induction of DT-diaphorase in human normal cells

The base level of DT-diaphorase activity in human bone marrow cells was very low, 1.2 ± 0.4 nmol MTT $min^{-1}$ $mg^{-1}$ protein; however, incubation with D3T resulted in a small, but significant increase in this activity to 12.7 ± 2.4 nmol MTT $min^{-1}$ $mg^{-1}$ protein ($P < 0.005$) (Table 3). The base level of enzyme activity in 293 human kidney cells was also very low, 2.2 ± 0.3 nmol MTT $min^{-1}$ $mg^{-1}$ protein, and D3T also increased DT-diaphorase activity in these cells to 7.7 ± 0.1 nmol MTT $min^{-1}$ $mg^{-1}$ protein ($P < 0.001$). In contrast, WI-38 human lung cells had an intermediate base level of DT-diaphorase activity, 76.5 ± 6.5 nmol MTT $min^{-1}$ $mg^{-1}$ protein, and this was increased to 182.3 ± 12.2 nmol MTT $min^{-1}$ $mg^{-1}$ protein ($P < 0.002$) by treatment with D3T.

### Combination treatment with D3T analogues and bioreductive anti-tumour agents

HL-60 cells were incubated with or without 50 μM Oltipraz or 50 μM D3T for 48 h and then with various concentrations of EO9 for 1 h. Cytotoxicity was determined by clonogenic assay (Figure 3). Treatment of HL-60 cells with 50 μM Oltipraz or 50 μM D3T increased DT-diaphorase activity from 3.3 ± 0.4 to 5.8 ± 0.4

© Cancer Research Campaign 1998

**Table 3** Induction of DT-diaphorase in human normal cells by D3T

| Cells | DT-diaphorase activity (nmol MTT $min^{-1}$ $mg^{-1}$ protein) | | *P* |
|---|---|---|---|
| | Control | D3T induced | |
| Bone marrow | 1.2 ± 0.4 | 12.7 ± 2.4 | < 0.005 |
| 293 Kidney | 2.2 ± 0.3 | 7.7 ± 0.1 | < 0.001 |
| WI-38 lung | 76.5 ± 6.5 | 182.3 ± 12.2 | < 0.002 |

Cells were incubated with or without 100 μM D3T at 37°C for 48 h. Cells were washed, pelleted, suspended in 100–200 μl of 0.25 M sucrose, sonicated and stored at –80°C. DT-diaphorase activity was measured as described in Materials and methods using menadione as the electron acceptor. The data represent the mean ± standard error of three or four determinations. Statistical significance was determined using a two-tailed *t*-test comparing the significance of the difference of the mean DT-diaphorase activity in control and D3T-treated cells.

($P < 0.001$) or 15.6 ± 1.5 ($P < 0.001$) nmol MTT $min^{-1}$ $mg^{-1}$ protein respectively. Pretreatment with Oltipraz enhanced the cytotoxicity of EO9 in HL-60 cells with the $D_0$ decreasing from 6.1 ± 0.5 μM to 4.3 ± 0.3 μM ($P < 0.02$). Pretreatment with D3T also increased the cytotoxicity of EO9 with the $D_0$ decreasing from 6.1 ± 0.5 μM to 3.3 ± 0.2 μM ($P < 0.001$), and this effect was significantly greater than the effect observed with Oltipraz ($P < 0.05$). The addition of the DT-diaphorase inhibitor, dicoumarol, before treatment with EO9 reversed the increased cytotoxicity with D3T ($P < 0.005$). In contrast, when 293 human normal kidney cells were treated with 50 μM D3T for 48 h, there was no significant increase in DT-diaphorase activity compared with control cells, and the cytotoxic activity of EO9 in these cells ($D_0$ = 31.7 ± 2.5 μM, determined by MTT assay) did not change significantly when the cells were pretreated with D3T (Figure 4).

H661 cells were incubated with or without 50 μM analogue 8 or 50 μM D3T for 48 h and then were treated with various concentrations of EO9 for 1 h. Cytotoxicity was determined by MTT assay (Figure 5). Treatment of H661 cells with 50 μM analogue 8 or 50 μM D3T increased DT-diaphorase activity from 168.4 ± 15.6 to 226.8 ± 17.9 ($P < 0.05$) or 266.7 ± 43.6 nmol MTT $min^{-1}$ $mg^{-1}$ protein respectively. Pretreatment with analogue 8 or D3T enhanced the cytotoxicity of EO9 in H661 cells with the $D_0$ decreasing from 0.7 ± 0.1 μM to 0.5 ± 0.1 0.1 μM ($P < 0.05$) or 0.5 ± 0.1 μM ($P < 0.05$), respectively, but the effect of D3T was not significantly different from that of analogue 8.

T47D human breast cancer cells were incubated with or without 75 μM D3T for 48 h and then were treated with various concentrations of MMC for 1 h. Cytotoxicity was determined by MTT assay (Figure 6). Incubation of the cells with D3T increased the level of DT-diaphorase activity from 25.8 ± 1.0 to 96.9 ± 5.6 nmol MTT $min^{-1}$ $mg^{-1}$ protein ($P < 0.001$) and also significantly enhanced the cytotoxicity of MMC by threefold. The $D_0$ decreased from 3.8 ± 0.1 μM for MMC alone to 1.2 ± 0.1 μM with D3T ($P < 0.001$).

### DISCUSSION

DT-diaphorase is a highly inducible enzyme that can be an important activating enzyme for bioreductive anti-tumour agents (Riley and Workman, 1992; Ross et al, 1993). The enzyme has also been shown to play an important role in detoxifying chemically reactive metabolities in cells, thus protecting the cell from their toxic and mutagenic effects (Beyer et al, 1988; Riley and Workman, 1992). Recent studies have investigated the use of inducers of DT-diaphorase in cancer prevention (Kelloff et al, 1990; Kensler et al, 1992; O'Dwyer et al, 1996). However, there have been few investigations of the potential use of inducers of DT-diaphorase to enhance the anti-tumour activity of anti-tumour agents that are activated by this enzyme. Shao et al (1995) found that the fish oil menhaden oil increased DT-diaphorase activity in an MX-1, human mammary carcinoma, tumour grown in athymic mice and also increased the response of the tumour xenograft to MMC. We have shown that D3T, an inducer of DT-diaphorase, can increase the level of DT-diaphorase activity in murine lymphoma cells without altering the level of enzyme activity in normal murine marrow cells, resulting in an increase in the anti-tumour activities of MMC and EO9 (Begleiter et al, 1996). In this study, we investigated the ability of D3T analogues to induce DT-diaphorase in different human tumour types and examined the ability of these analogues to enhance the cytotoxic activity of the bioreductive agents EO9 and MMC in human tumours.

The results of this study demonstrated that D3T can increase the level of DT-diaphorase activity in most human tumour cell types. Overall, 28 of 38 tumour cell lines showed significant increases in enzyme activity after treatment with 100 μM D3T that ranged from 1.2- to 8.0-fold, or from 2.5 to 590 nmol MTT $min^{-1}$ $mg^{-1}$ protein in absolute terms (Table 1). Leukaemia, lung, colon, stomach, breast, ovary, prostate and melanoma tumour cells lines were all inducible. In contrast, three head and neck tumours and one liver tumour were not induced by D3T. In addition, some tumour types appeared to be more readily induced. For example, all the leukaemia cell lines were induced, as were all the gastric tumour, ovarian tumour and malignant melanoma cell lines. However, the differences in inducibility of DT-diaphorase activity in the different cell types may simply reflect the particular cell lines of the tumour types examined in this study.

The mechanisms responsible for the differences in DT-diaphorase induction in different cell lines are unknown. Regulation of expression of DT-diaphorase activity is complex and may include regulation of transcription (Jaiswal, 1991; Belinsky and Jaiswal, 1993; Yao and O'Dwyer, 1995; Wang and Williamson, 1996), alternative splicing of DT-diaphorase mRNA (Gasdaska et al, 1995; Hu et al, 1996; Yao et al, 1996) and the presence of a polymorphism due to a mutation at position 609 of

 © Cancer Research Campaign 1998

**Figure 4** Effect of D3T on cytotoxic activity of EO9 and on the DT-diaphorase activity in 293, human normal kidney cells. Cells were incubated at 37°C with or without 50 μM D3T for 48 h. Cells were then treated with various concentrations of EO9 for 1 h. Surviving cell fraction was determined by MTT assay (Johnston et al, 1994). The points represent the mean surviving cell fraction ± standard error of five determinations. The lines are linear regression lines. Inset, level of DT-diaphorase activity in control and cells treated with D3T. The bars represent the mean DT-diaphorase activity ± standard error of four determinations. The means were compared using a *t*-test evaluating the significance of the difference of the DT-diaphorase activity in the control and D3T-treated cells

**Figure 5** Effect of analogue 8 or D3T on cytotoxic activity of EO9 and DT-diaphorase activity in H661, human large-cell lung tumour cells. Cells were incubated at 37°C with or without 50 μM analogue 8 or 50 μM D3T for 48 h. Cells were then treated with various concentrations of EO9 for 1 h. Surviving cell fraction was determined by MTT assay (Johnston et al, 1994). The points represent the mean surviving cell fraction ± standard error of seven to nine determinations. The lines are linear regression lines. Inset, level of DT-diaphorase activity in control and cells treated with analogue 8 or D3T. The bars represent the mean DT-diaphorase activity ± standard error of four determinations. The means were compared using a *t*-test evaluating the significance of the difference of the DT-diaphorase activity in the control and D3T analogue-treated cells the $NQO_1$cDNA (Traver et al, 1992; Kuehl et al, 1995) that results in a protein with very low enzyme activity (Traver et al, 1997). Whether any of these mechanisms are responsible for the different effects of the D3T analogues on DT-diaphorase activity in the different human cell lines studied remains to be determined.

There was no obvious relationship between the base level of DT-diaphorase activity in the tumour cells and the ability of D3T to increase enzyme activity. Although four of the ten tumour cell lines that were not induced by D3T had high base levels of DT-diaphorase activity, all the melanoma tumours, and other cell lines, that had high base levels of enzyme activity were induced. While the increase in enzyme activity relative to the base level was low in these cells, the absolute increase in enzyme activity was very high. In addition, the oestrogen or androgen receptor status of the cells did not appear to affect the induction of DT-diaphorase. Of the six breast cancer cell lines that were induced by D3T, three were oestrogen receptor positive and three were oestrogen receptor negative. Similarly, one of the two prostate cell lines that were induced by D3T was androgen receptor positive while the other was androgen receptor negative.

DT-diaphorase induction by D3T and 12 analogues was examined in five different tumour tissue types to identify any tissue-specific DT-diaphorase induction profiles. Overall, the D3T analogues did not appear to show any obvious tissue specificity for enzyme induction. However, some of the D3T analogues were better inducers of DT-diaphorase than others in the human tumour cell lines examined, and this may indicate some structure–activity relationships. The parent compound, D3T, was the most consistent inducer, producing significant increases in enzyme activity in six of the eight cell lines. Analogues 4, 5, 7, 8 and 9 were also consistent inducers of DT-diaphorase activity, producing significant increases in enzyme activity in five cell lines. Of these, D3T and analogues 7, 8, and 9 generally produced the largest increases in DT-diaphorase activity. In contrast, analogue 10 was the poorest inducer in the cell lines studied. These results suggest that the parent D3T is the best inducer of DT-diaphorase activity of the analogues studied. However, the carbamoyl group in analogue 7, the S-oxide in analogue 8 and the ring-opened structure in analogue 9, in some instances, may produce greater increases in DT-diaphorase activity than D3T. These studies do not indicate any apparent difference in induction capacity between analogues with substituents at position 4 or 5 of the D3T ring, but the salt structure in analogue 10 may reduce induction capacity, possibly as a result of decreased cellular uptake.

Talalay (1989) reviewed the structural requirements for induction of DT-diaphorase by monofunctional inducers and postulated that the inducers are all Michael reaction acceptors, characterized

© Cancer Research Campaign 1998

**Figure 6** Effect of D3T on cytotoxic activity of MMC and DT-diaphorase activity in T47D, human breast cancer cells. Cells were incubated at 37°C with or without 75 μM D3T for 48 h. Cells were then treated with various concentrations of MMC for 1 h. Surviving cell fraction was determined by MTT assay (Johnston et al, 1994). The points represent the mean surviving cell fraction ± standard error of three to seven determinations. The lines are linear regression lines. Inset, level of DT-diaphorase activity in control and cells treated with D3T. The bars represent the mean DT-diaphorase activity ± standard error of 6–11 determinations. The means were compared using a *t*-test evaluating the significance of the difference of the DT-diaphorase activity in the control and D3T analogue-treated cells by olefinic or acetylenic linkages that are rendered electrophilic by conjugation with electron-withdrawing groups. Prestera et al (1993) also surveyed a list of DT-diaphorase inducers, including D3T, and the only apparent universal property was their capacity for reaction with sulphydryls by either oxidoreduction or alkylation. Structural modifications of D3T may, therefore, alter the electrophilic character of the analogues or their ability to react with sulphydryls.

Egner et al (1994) compared the induction of DT-diaphorase in Hepa 1c1c7, mouse hepatoma cells, by 25 D3T analogues. This group of analogues included D3T, Oltipraz, analogues 1–6, and analogue 8, which we also examined in our study. Consistent with our findings, D3T was one of the more potent DT-diaphorase inducers in the Hepa 1c1c7 cells. In addition, the 5-ethyl analogue (analogue 4) and the S-oxide analogue (analogue 8) were relatively potent inducers in both studies. In contrast, the 4-ethyl analogue (analogue 3), the 4-phenyl analogue (analogue 6) and the 5-t-butyl analogue (analogue 5) were good inducers in Hepa 1c1c7 cells but poor inducers in the human tumour cell lines. Furthermore, the ring-opened analogue (analogue 9) was a good inducer of DT-diaphorase in human tumours, but a ring-opened analogue of Oltipraz was inactive in the mouse hepatoma cells. Comparison of the results of these two studies indicate both similarities and differences in induction of DT-diaphorase by D3T analogues in human and mouse tumour cells. Whether these relate to species differences or differences in tumour type is unknown.

The base levels of DT-diaphorase activity were very low in the normal marrow and kidney cells that we examined. While D3T produced significant increases in enzyme activity in these cells, the actual increases were only 11.5 and 5.5 nmol MTT min$^{-1}$ mg$^{-1}$ protein, respectively, less than half the increase observed in HL-60 leukaemia cells. In contrast, D3T increased the level of DT-diaphorase activity in normal human lung cells by 2.4-fold.

Combination treatment with Oltipraz or D3T and EO9 increased the anti-tumour activity of EO9 in HL-60, human promyelocytic leukaemia cells (Figure 3). Oltipraz and D3T increased the level of DT-diaphorase activity in these cells by 1.7- and 4.7-fold, respectively ($P < 0.001$), and also increased the cytotoxic activity of EO9 by 1.4- and 1.8-fold, respectively, in these cells ($P < 0.02$). The increased cytotoxic activity was due to the elevated DT-diaphorase activity, as dicoumarol, an inhibitor of the enzyme, reversed the effect of D3T on EO9 cytotoxic activity ($P < 0.005$). In addition, D3T did not increase the activities of NADPH:cytochrome P450 reductase, NADH:cytochrome $b_5$ reductase or xanthine dehydrogenase, other enzymes that can activate bioreductive agents (Pan et al, 1984; Gustafson and Pritsos, 1992; Hodnick and Sartorelli, 1993), in these cells.

Pretreatment of H661, human non-small-cell lung cancer cells with analogue 8 or D3T significantly increased the cell kill observed with EO9 by 1.5- and 1.6-fold respectively ($P < 0.05$) (Figure 5). The enhancement of EO9 cytotoxicity by the D3T analogues parallelled their effect on induction of DT-diaphorase. Thus, other D3T analogues that produce greater induction of DT-diaphorase activity may further enhance the cytotoxic activity of bioreductive agents. Similarly, combination treatment with D3T and MMC increased the anti-tumour activity of MMC in T47D, human breast carcinoma cells (Figure 6). D3T increased the level of DT-diaphorase activity in these cells by 3.8-fold ($P < 0.001$) and also increased the cytotoxic activity of MMC by threefold in these cells ($P < 0.001$).

The major toxicities observed with MMC and EO9 are bone marrow and kidney toxicity respectively (Hortobagyi, 1993; Schellens et al, 1994). Treatment with 100 μM D3T produced small, but significant, increases in the levels of DT-diaphorase activity in normal human marrow and kidney cells. Because the increases in DT-diaphorase activities were very small in these cells, it is unlikely that the toxicity to these cells would be significantly increased. Indeed, 50 μM D3T did not effect the toxicity of EO9 in the human normal kidney cells, but this concentration of D3T did not significantly increase DT-diaphorase activity in the kidney cells. Furthermore, we have shown previously that D3T did not increase the toxicity of MMC to mouse marrow cells (Begleiter et al, 1996). In contrast, the increase in enzyme activity in the normal human lung cells was considerably larger. This effect might increase MMC toxicity to normal lung tissues in a clinical setting. However, MMC has been shown to produce pulmonary fibrosis in approximately 5% of patients (Klein and Wilds, 1983) by a mechanism that probably involves redox cycling and the formation of reactive oxygen species. Thus, it is possible that the increase in DT-diaphorase activity in normal lung cells with D3T treatment may serve to decrease this lung toxicity, as two-electron reduction of MMC to its hydroquinone by DT-diaphorase would decrease reactive oxygen species formation.

© Cancer Research Campaign 1998

The overall effect of DT-diaphorase induction on MMC toxicity in the lung will need to be assessed in an in vivo study.

These studies do not provide any conclusive evidence about the level of increase in DT-diaphorase activity that is required to increase the cytotoxic activity of the bioreductive anti-tumour agents. It is not known whether there is a threshold of induction that must be exceeded before the cytotoxic activity of the bioreductive agents is increased. For example, an increase in DT-diaphorase activity of 2.5 nmol MTT $min^{-1}$ $mg^{-1}$ protein in HL-60, human leukaemia cells, produced by 50 μM D3T, increased cell kill by EO9 by 1.4-fold, while an increase in enzyme activity of 1.1 nmol MTT $min^{-1}$ $mg^{-1}$ protein in 293, human normal kidney cells, also produced by 50 μM D3T, had no effect on EO9 activity in these cells. If there is a threshold level of DT-diaphorase induction required for an effect on the activity of bioreductive agents, it is still unclear whether this level will be the same for all cells or, as is more likely, the level will be different in different cells. Furthermore, it is not known whether the absolute level of increase in DT-diaphorase activity or the level of increase relative to the base level of enzyme activity is most important for determining the effect on the cytotoxic activity of the anti-tumour agents. Despite these ambiguities, this study demonstrates that non-toxic concentrations of D3T analogues can produce large increases in DT-diaphorase activity in a wide variety of human tumour cells and can enhance the anti-tumour activity of EO9 and MMC with only small effects on normal kidney and marrow cells, which represent the most important sites of clinical toxicity to these bioreductive agents.

The GSTs are a family of phase II detoxifying enzymes that have been shown to be coordinately induced with DT-diaphorase in some tissues (Talalay, 1989). These enzymes may also protect cells from the toxic and mutagenic effects of foreign chemicals (Prestera et al, 1993). In addition, GSTs have been shown to play an important role in resistance to a variety of anti-tumour agents, including MMC, by aiding in the removal of the drugs from cells (Waxman, 1990; Xu et al, 1994). Thus, if D3T were to increase the levels of both DT-diaphorase and GST activity in tumour cells, this might not result in a net increase in anti-tumour activity. This did not occur in our studies as we did observe an increase in anti-tumour activity with both EO9 and MMC in three tumour cell lines. Furthermore, we did not see any increase in GST activity in HL-60 cells treated with D3T, as has been observed previously (Li et al, 1994). Therefore, it appears that it is possible to increase DT-diaphorase activity in tumour cells without increasing GST. Additional studies are required to determine whether this effect is observed in other tumour cells, in normal tissues or with other inducers.

In summary, we have shown that D3T analogues significantly increased the level of DT-diaphorase activity in 28 of 38 human tumour cells representing a wide variety of tumour types. DT-diaphorase activity was also increased in normal human bone marrow and kidney cells, but the increases were small in these cases. D3T produced a significant increase in enzyme activity in normal human lung cells. Modifications to the basic D3T structure, such as the 5-carbamoyl substituent, the S-oxide function, or opening of the dithiolethione ring may increase the enzyme inductive capacity. D3T analogues that combine these structural features may prove to be better inducers of DT-diaphorase. Additional studies are required to identify D3T analogues that produce the optimum selective increase in DT-diaphorase activity in tumour cells compared with normal cells.

Combination treatment of human tumour cells with D3T analogues and the bioreductive anti-tumour agents, MMC or EO9, produced significant increases in cytotoxic activity in human tumour cells, and the level of enhancement of anti-tumour activity paralleled the level of DT-diaphorase induction. In contrast, D3T did not effect the toxicity of EO9 in normal kidney cells. Thus, it may be possible to use inducers of DT-diaphorase to enhance the effectiveness of bioreductive anti-tumour agents that are activated by this enzyme. This approach appears to be applicable to different agents and in different tumour cells. The development of D3T analogues that are more potent and selective inducers of DT-diaphorase would increase the potential use of this therapeutic approach. Additional studies with other inducers, anti-tumour agents and cells are required to identify the optimum uses for this new treatment strategy.

### ACKNOWLEDGEMENTS

We are grateful to the Medical Research Council of Canada, Manitoba Cancer Treatment and Research Foundation, Manitoba Health Research Council, the University of Manitoba, and the National Cancer Institute, USA (CA39416), for financial support. GPD and XW are recipients of George H Sellers Studentships.

### REFERENCES

Atallah AS, Landolph JR, Ernster L and Hochstein P (1988) DT-diaphorase activity and the cytotoxicity of quinones in C3H/10T1/2 mouse embryo cells. *Biochem Pharmacol* **37**: 2451–2459

Barham HM, Inglis R, Chinje EC and Stratford IA (1996) Development and validation of a spectrophotometric assay for measuring the activity of NADH: cytochrome $b_5$ reductase in human tumor cells. *Br J Cancer* **74**: 1188–1193

Beall HD, Liu YF, Siegel D, Bolton EM, Gibson NW and Ross D (1996) Role of NAD(P)H:quinone oxidoreductase (DT-diaphorase) in cytotoxicity and induction of DNA damage by streptonigrin. *Biochem Pharmacol* **51**: 645–652

Begleiter A and Leith MK (1990) Activity of quinone alkylating agents in quinone-resistant cells. *Cancer Res* **50**: 2872–2876

Begleiter A and Leith MK (1995) Induction of DT-diaphorase by doxorubicin and combination therapy with mitomycin C in vitro. *Biochem Pharmacol* **50**: 1281–1286

Begleiter A, Robotham E, Lacey G and Leith MK (1989) Increased sensitivity of quinone resistant cells to mitomycin C. *Cancer Lett* **45**: 173–176

Begleiter A, Robotham E and Leith MK (1992) Role of NAD(P)H:(quinone acceptor)oxidoreductase (DT-diaphorase) in activation of mitomycin C under hypoxia. *Mol Pharmacol* **41**: 677–682

Begleiter A, Leith MK and Curphey TJ (1996) Induction of DT-diaphorase by 1,2-dithiole-3-thione and increase of antitumour activity of bioreductive agents. *Br J Cancer* **74** (suppl. 27): S9–S14

Belcourt MF, Hodnick WF, Rockwell S and Sartorelli AC (1996) Differential toxicity of mitomycin C and porfiromycin to aerobic and hypoxic Chinese hamster ovary cells overexpressing human NADPH:cytochrome c (P-450) reductase. *Proc Natl Acad Sci USA* **93**: 456–460

Belinsky M and Jaiswal AK (1993) NAD(P)H:quinone oxidoreductase1 (DT-diaphorase) expression in normal and tumor tissues. *Cancer Metastasis Rev* **12**: 103–117

Benson AM, Hunkeler MJ and Talalay P (1980) Increase of NAD(P)H:quinone reductase by dietary antioxidants: possible role in protection against carcinogenesis and toxicity. *Proc Natl Acad Sci USA* **77**: 5216–5220

Beyer RE, Segura Aguilar JE and Ernster L (1988) The anticancer enzyme DT-diaphorase is induced selectively in liver during ascites hepatoma growth. *Anticancer Res* **8**: 233–238

Bueding E, Dolan P and Leroy JP (1982) The antischistosomal activity of oltipraz. *Res Commun Chem Pathol Pharmacol* **37**: 293–303

Egner PA, Kensler TW, Prestera T, Talalay P, Libby AH, Joyner HH and Curphey TJ (1994) Regulation of phase 2 enzyme induction by oltipraz and other dithiolethiones. *Carcinogenesis* **15**: 177–181

Fitzsimmons SA, Workman P, Grever M, Paull K, Camalier R and Lewis AD (1996) Reductase enzyme expression across the National Cancer Institute tumor cell line panel: correlation with sensitivity to mitomycin C and EO9. *J Natl Cancer Inst* **88**: 259–269

Gasdaska PY, Fisher H and Powis G (1995) An alternatively spliced form of NQO1 (DT-diaphorase) messenger RNA lacking the putative quinone substrate binding site is present in human normal and tumor tissues. *Cancer Res* **55**: 2542–2547

Gustafson DL and Pritsos CA (1992) Bioactivation of mitomycin C by xanthine dehydrogenase from EMT6 mouse mammary carcinoma tumors. *J Natl Cancer Inst* **84**: 1180–1185

Gustafson DL, Swanson JD and Pritsos CA (1991) Role of xanthine oxidase in the potentiation of doxorubicin-induced cardiotoxicity by mitomycin C. *Cancer Commun* **3**: 1–6

Habig WH, Pabst MJ and Jakoby WB (1974) Glutathione S-transferase, The first enzymatic step in mercaptouric acid formation. *J Biol Chem* **249**: 7130–7139

Hodnick WF and Sartorelli AC (1993) Reductive activation of mitomycin C by NADH:cytochrome b5 reductase. *Cancer Res* **53**: 4907–4912

Hortobagyi GN (1993) Mitomycin: its evolving role in the treatment of breast cancer. *Oncol* **50** (suppl. 1): 1–8

Hu LT, Stamberg J and Pan SS (1996) The NAD(P)H:quinone oxidoreductase locus in human colon carcinoma HCT 116 cells resistant to mitomycin C. *Cancer Res* **56**: 5253–5259

Jaiswal AK (1991) Human NAD(P)H:quinone oxidoreductase (NQO-1) gene structure and induction by dioxin. *Biochemistry* **30**: 10647–10653

Jaiswal AK, Burnett P, Adesnick M and McBride OW (1990) Nucleotide and deduced amino acid sequence of a human cDNA (NQO2) corresponding to a second member of the NAD(P)H:quinone oxidoreductase gene family. Extensive polymorphism at the NQO2 gene locus on chromosome 6. *Biochemistry* **29**: 1899–1906

Johnston JB, Verburg L, Shore T, Williams M, Israels LG and Begleiter A (1994) Combination therapy with nucleoside analogs and alkylating agents. *Leukemia* **8**(suppl.): S140–S143

Kelloff GJ, Malone WF, Boone CW, Sigman CC and Fay JR (1990) Progress in applied chemoprevention research. *Semin Oncol* **17**: 438–455

Kensler TW and Helzlsouer KJ (1995) Oltipraz: clinical opportunities for cancer chemoprevention. *J Cell Biochem* suppl. 22: 101–107

Kensler T, Styczynski P, Groopman J, Helzlsouer K, Curphey T, Maxuitenko Y and Roebuck BD (1992) Mechanisms of chemoprevention by oltipraz. *J Cell Biochem* suppl. 16I: 167–172

Klein DS and Wilds PR (1983) Pulmonary toxicity of antineoplastic agents: anaesthetic and postoperative implications. *Can Anaesth Soc J* **30**: 399–405

Kuehl BL, Paterson JWE, Peacock JW, Paterson MC and Rauth AM (1995) Presence of a heterozygous substitution and its relationship to DT-diaphorase activity. *Br J Cancer* **72**: 555–561

Li Y, Lafuente A and Trush MA (1994) Characterization of quinone reductase, glutathione and glutathione S-transferase in human myeloid cell lines: induction by 1,2-dithiole-3-thione and effects on hydroquinone-induced cytotoxicity. *Life Sci* **54**: 901–916

Malkinson AM, Siegel D, Forrest GL, Gazdar AF, Oie HK, Chan DC, Bunn PA, Mabry M, Dykes DJ, Harrison SD and Ross D (1992) Elevated DT-diaphorase activity and messenger RNA content in human non-small lung carcinoma: relationship to the response of lung xenografts to mitomycin C. *Cancer Res* **52**: 4752–4757

Maxuitenko YY, Curphey TJ, Kensler TW and Roebuck BD (1996) Protection against aflatoxin $B_1$-induced hepatic toxicity as a short-term screen of cancer chemopreventive dithiolethiones. *Fund Appl Toxicol* **32**: 250–259

Mikami K, Naito M, Tomida A, Yamada M, Sirakusa T and Tsuruo T (1996) DT-diaphorase as a critical determinant of sensitivity to mitomycin C in human colon and gastric carcinoma cell lines. *Cancer Res* **56**: 2823–2826

Nishiyama M, Saeki S, Aogi K, Hirabayashi N and Toge T (1993) Relevance of DT-diaphorase activity to mitomycin C (MMC) efficacy on human cancer cells: differences in in vitro and in vivo systems. *Int J Cancer* **53**: 1013–1016

O'Dwyer PJ, Szarka CE, Yao K-S., Halbherr TC, Pfeiffer GR, Green F, Gallo JM, Brennan J, Frucht H, Goosenberg EB, Hamilton TC, Litwin S, Balshem AM, Engstrom PF and Clapper ML (1996) Modulation of gene expression in subjects at risk for colorectal cancer by the chemopreventative dithiolethione Oltipraz. *J Clin Invest* **98**: 1210–1217

Pan SS, Andrews PA, Glover CJ and Bachur NR (1984) Reductive activation of mitomycin C and mitomycin C metabolites catalyzed by NADPH-cytochrome P-450 reductase and xanthine oxidase. *J Biol Chem* **259**: 959–966

Patterson AV, Robertson N, Houlbrook S, Stephens MA, Adams GE, Harris AL, Stratford IJ and Carmichael J (1994) The role of DT-diaphorase in determining the sensitivity of human tumor cells to tirapazamine (SR 4233). *Int J Radiat Oncol Biol Phys* **29**: 369–372

Plumb JA, Gerritsen M, Milroy R, Thomson P and Workman P (1994) Relative importance of DT-diaphorase and hypoxia in the bioactivation of EO9 by human lung tumor cell lines. *Int J Radiat Oncol Biol Phys* **29**: 295–299

Powis G, Gasdaska PY, Gallegos A, Sherrill K and Goodman D (1995) Over-expression of DT-diaphorase in transfected NIH 3T3 cells does not lead to increased anticancer quinone drug sensitivity: a questionable role for the enzyme as a target for bioreductively activated anticancer drugs. *Anticancer Res* **15**: 1141–1145

Prestera T, Zhang Y, Spencer SR, Wilczak CA and Talalay P (1993) The electrophile counterattack response: protection against neoplasia and toxicity. *Adv Enzyme Regul* **33**: 281–296

Prochaska HJ and Santamaria AB (1988) Direct measurement of NAD(P)H:quinone reductase from cells cultured in microtiter wells: a screening assay for anticarcinogenic enzyme inducers. *Anal Biochem* **169**: 328–336

Riley RJ and Workman P (1992) DT-diaphorase and cancer chemotherapy. *Biochem Pharmacol* **43**: 1657–1669

Ross D, Siegel D, Beall H, Prakash AS, Mulcahy RT and Gibson NW (1993) DT-diaphorase in activation and detoxification of quinones. *Cancer Metastasis Rev* **12**: 83–101

Schellens JHM, Planting AST, Van Acker BAC, Loos WJ, De Boer-Dennert M, Van der Burg MEL, Koier I, Krediet RT, Stoter G and Verweij J (1994) Phase I and pharmacologic study of the novel indoloquinone bioreductive alkylating cytotoxic drug E09. *J Natl Cancer Inst* **86**: 906–912

Schlager JJ and Powis G (1990) Cytosolic NAD(P)H:(quinone-acceptor)oxidoreductase in human normal and tumour tissue: effects of cigarette smoking and alcohol. *Int J Cancer* **45**: 403–409

Shao Y, Pardini L and Pardini RS (1995) Dietary menhaden oil enhances mitomycin C antitumor activity toward human mammary carcinoma MX-1. *Lipids* **30**: 1035–1045

Siegel D, Gibson NW, Preusch PC and Ross D (1990) Metabolism of diaziquone by NAD(P)H:(quinone acceptor) oxidoreductase (DT-diaphorase): role in diaziquone-induced DNA damage and cytotoxicity in human colon carcinoma cells. *Cancer Res* **50**: 7293–7300

Smitskamp-Wilms EG, Giaccone G, Pinedo HM, Van der Laan BFAM and Peters GJ (1995) DT-diaphorase activity in normal and neoplastic human tissues: an indicator for sensitivity to bioreductive agents? *Br J Cancer* **72**: 917–921

Strobel HW and Dignam JD (1978) Purification and properties of NADPH-cytochrome P-450 reductase. *Methods Enzymol* **52**: 89–96

Talalay P (1989) Mechanisms of induction of enzymes that protect against chemical carcinogenesis. *Adv Enzyme Regul* **28**: 237–250

Traver RD, Horikoshi T, Danenberg KD, Stadlbauer TH, Danenberg PV, Ross D and Gibson NW (1992) NAD(P)H:quinone oxidoreductase gene expression in human colon carcinoma cells: characterization of a mutation which modulates DT-diaphorase activity and mitomycin sensitivity. *Cancer Res* **52**: 797–802

Traver RD, Siegel D, Beall HD, Phillips RM, Gibson NW, Franklin WA and Ross D (1997) Characterization of a polymorphism in NAD(P)H:quinone oxidoreductase (DT-diaphorase). *Br J Cancer* **75**: 69–75

Wang B and Williamson G (1996) Transcriptional regulation of the human NAD(P)H:quinone oxidoreductase ($NQO_1$) gene by monofunctional inducers. *Biochim Biophys Acta* **1307**: 104–110

Waxman DJ (1990) Glutathione S-transferases: role in alkylating agent resistance and possible target for modulation chemotherapy – a review. *Cancer Res* **50**: 6449–6454

Workman P and Stratford IJ (1993) The experimental development of bioreductive drugs and their role in cancer therapy. *Cancer Metastasis Rev* **12**: 73–82

Yao KS and O'Dwyer PJ (1995) Involvement of NF-kappaB in the induction of NAD(P)H:quinone oxidoreductase (DT-diaphorase) by hypoxia, oltipraz and mitomycin. *Biochem Pharmacol* **49**: 275–282

Yao KS, Godwin AK, Johnson C and O'Dwyer PJ (1996) Alternative splicing and differential expression of DT-diaphorase transcripts in human colon tumors and in peripheral mononuclear cells in response to mitomycin C treatment. *Cancer Res* **56**: 1731–1736

Xu BH, Gupta V and Singh SV (1994) Characterization of a human bladder cancer cell line selected for resistance to mitomycin C. *Int J Cancer* **58**: 686–692

© Cancer Research Campaign 1998